# Large-scale correlation network construction for unraveling the coordination of complex biological systems

**DOI:** 10.1038/s43588-023-00429-y

**Published:** 2023-04-13

**Authors:** Martin Becker, Huda Nassar, Camilo Espinosa, Ina A. Stelzer, Dorien Feyaerts, Eloise Berson, Neda H. Bidoki, Alan L. Chang, Geetha Saarunya, Anthony Culos, Davide De Francesco, Ramin Fallahzadeh, Qun Liu, Yeasul Kim, Ivana Marić, Samson J. Mataraso, Seyedeh Neelufar Payrovnaziri, Thanaphong Phongpreecha, Neal G. Ravindra, Natalie Stanley, Sayane Shome, Yuqi Tan, Melan Thuraiappah, Maria Xenochristou, Lei Xue, Gary Shaw, David Stevenson, Martin S. Angst, Brice Gaudilliere, Nima Aghaeepour

**Affiliations:** 1 Department of Anesthesiology, Perioperative and Pain Medicine, Stanford University School of Medicine, Palo Alto, CA, USA; 2 Department of Pediatrics, Stanford University School of Medicine, Palo Alto, CA, USA; 3 Department of Biomedical Data Science, Stanford University School of Medicine, Palo Alto, CA, USA; 4 Department of Computer Science and Electrical Engineering, University of Rostock, Rostock, Germany; 5 Department of Pathology, Stanford University School of Medicine, Palo Alto, CA, USA; 6 These authors contributed equally: Martin Becker, Huda Nassar, Camilo Espinosa

## Abstract

Advanced measurement and data storage technologies have enabled high-dimensional profiling of complex biological systems. For this, modern multiomics studies regularly produce datasets with hundreds of thousands of measurements per sample, enabling a new era of precision medicine. Correlation analysis is an important first step to gain deeper insights into the coordination and underlying processes of such complex systems. However, the construction of large correlation networks in modern high-dimensional datasets remains a major computational challenge owing to rapidly growing runtime and memory requirements. Here we address this challenge by introducing CorALS (Correlation Analysis of Large-scale (biological) Systems), an open-source framework for the construction and analysis of large-scale parametric as well as non-parametric correlation networks for high-dimensional biological data. It features off-the-shelf algorithms suitable for both personal and high-performance computers, enabling workflows and downstream analysis approaches. We illustrate the broad scope and potential of CorALS by exploring perspectives on complex biological processes in large-scale multiomics and single-cell studies.

The advancement of modern technologies, including single-cell^1^ and multiomics approaches^2^, wearable devices^3^, and integrated electronic health records^4,5^, have enabled an exciting era of precision medicine. These technologies regularly produce datasets with hundreds of thousands of variables (here referred to as features), allowing for unprecedented profiling of complex biological processes such as diseases, pregnancy or healing^2,6,7^. Correlation analysis is typically the first step to gain insights into a complex system (56% of all papers on the preprint server bioRxiv contain the word ‘correlation’). While erroneous data may skew correlation analyses^8^ and correlation does not imply causation, correlation analysis can guide coordinated subprocesses in complex systems for further investigation. Consequently, a broad range of algorithms have been developed for analyzing large-scale correlation networks from the perspective of topology, connectivity patterns or community structures^9,10^. In addition, extensive gene graphs and cell-to-cell relations derived from large-scale correlation networks are integrated in modern deep learning and graph neural network applications^11,12^.

Despite diverse applications, the construction of correlation networks for large datasets remains a major computational challenge (for example, for only n=1,000 features, at least 499,000 pairs need to be examined). As such, computation time and memory requirements for constructing correlation networks grow rapidly and quickly exceed computational resources as the dimensionality of the datasets increases. Current approaches for constructing correlation networks (Supplementary Section 5) either rely on specialized parallel processing and high-performance-computing frameworks (for example, graphics processing units, MapReduce and so)^13–17^, focus on specialized correlation measure^18,19^ or address only limited aspects of time and memory requirements^20–23^.

Thus, here we introduce an open-source framework for high-dimensional correlation analysis called CorALS (Correlation Analysis of Large-scale (biological) Systems). CorALS enables efficient correlation computation, as well as top-*k* and differential correlation network approximations without requiring specialized software or hardware. We provide CorALS as an easy-to-use and extensible Python package. The corresponding routines are notably faster and require substantially less memory than commonly employed methods, allowing for complex analyses even on regular laptop computers. To achieve this, CorALS combines specialized vector projections, modern optimized linear algebra routines, spatial partitioning techniques and big data programming models (Fig. 1). CorALS supports Pearson correlation, the non-parametric Spearman correlation and the Phi coefficient for binary variables. We demonstrate the broad scope and potential of CorALS by analyzing several high-dimensional datasets, and present complementary perspectives on large-scale multiomics and single-cell datasets, revealing concerted coordination of biological systems during pregnancy. Overall, CorALS will allow practitioners to integrate large-scale correlation network analysis into rapid turnaround workflows that have previously been inaccessible owing to time and resource limitations, and enable the development of downstream applications for deeper insights into complex systems.

## Results

**Fig. 1 |:**
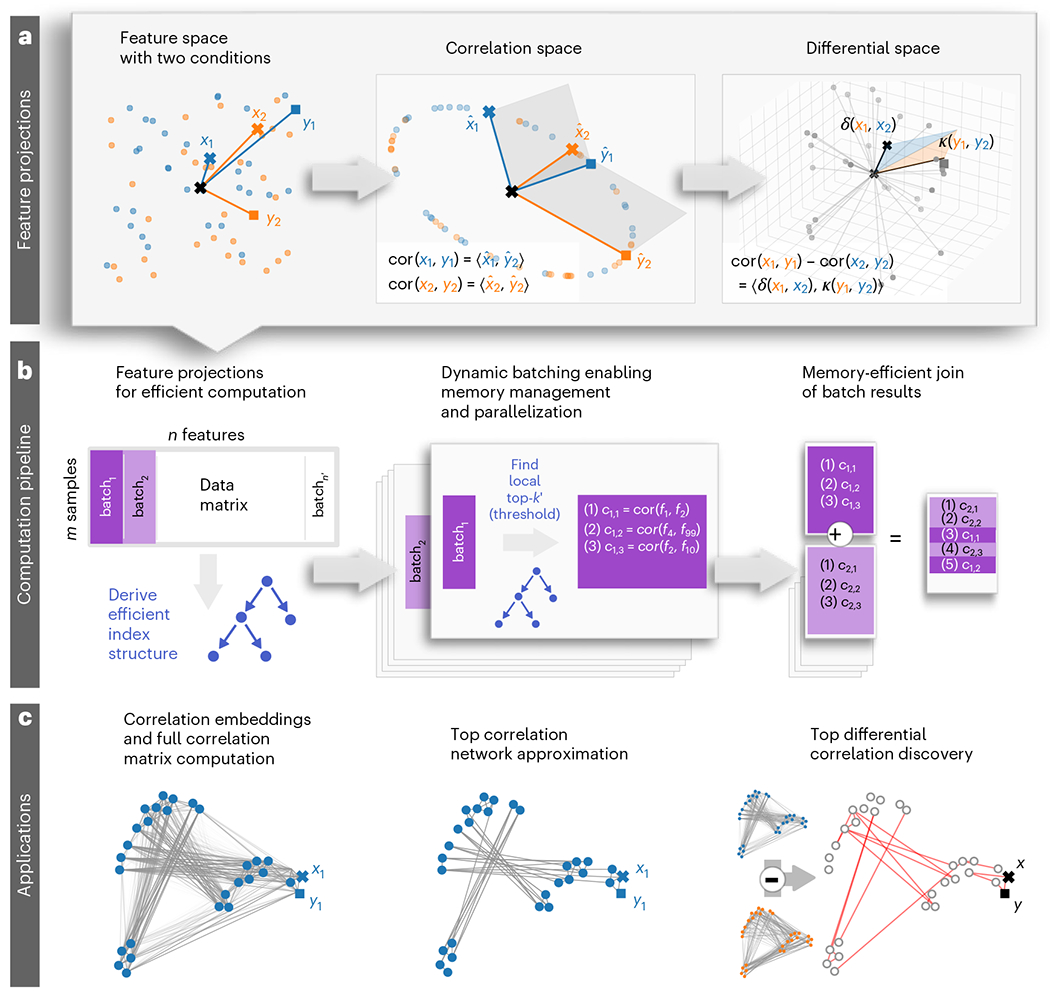
Overview of the CorALS framework. **a-c**, CorALS leverages feature projections into specialized vector spaces (**a**) embedded into a flexible computational pipeline (**b**) for large-scale correlation analysis (**c**). In particular, CorALS exploits the direct connection between Euclidean distance and the correlation of individual features in correlation space (**a**, middle), as well as the Euclidean distance and correlation differences of feature pairs across conditions in differential space (**a**, right), to derive efficient indexing structures (**b**, left). These indexes are utilized in a computational pipeline that splits correlation computations into batches based on a specifically designed approximation scheme for effective memory management and parallelization (**b**, middle). Batches are then joined in a memory efficient manner to yield the final correlation results (**b**, right). This enables applications such as full correlation matrix computation and correlation-based feature embeddings (**c**, left), top correlation network approximations (**c**, middle) and differential correlation discovery (**c**, right) for large-scale, high-dimensional datasets. Points represent features (two specific features are denoted as x and ), and subindices and colors indicate two conditions (1, blue; 2 , orange). $x_{1}$ and $x_{2}$ (and $y_{1}$ and $y_{2}$ ) are the same feature illustrated by a cross (square) marker across the two conditions. In a, feature projections are denoted as $\overset{ˆ}{•}$ in the middle panel, and δ and κ in the right panel. In b, individual features are represented as $f_{i}$, and $c_{i,j}$ is a short notation for the correlation between feature $f_{i}$ and $f_{j}$.

CorALS enables efficient, large-scale correlation analysis for high-dimensional data by employing a combination of versatile vector projections and big data computation models. In particular, features (Fig. 1a, left) are projected onto correlation vectors (Fig. 1a, middle) or embedded into a differential space (Fig. 1a, right). On the basis of these feature projections, CorALS exploits the tight relation of the scalar product and Euclidean distance in the corresponding vector space to derive efficient indexing schemes (Fig. 1b, left). These index structures are then embedded into a computational pipeline that splits correlation analysis tasks into batches (Fig. 1b, middle), which yield intermediate results based on a specifically designed approximation scheme. This scheme ensures that aggregating intermediate top-*k* results yield accurate approximations of the global correlation network (Fig. 1b, right). Importantly, the batched approach allows for effective memory management and inherent parallelization. Further technical details can be found in ‘Efficient approximation of correlation networks’ in Methods. On the basis of this framework, CorALS enables a wide variety of efficient analytical components (Fig. 1c). The following sections introduce these components and illustrates their runtime and memory advantages. For this, we employ several real-world high-dimensional datasets, in the context of pregnancy-related disease (pre-eclampsia), healthy pregnancy and cancer^1,6,24–26^. See Table 1 for basic dataset statistics and ‘Datasets’ in Methods for more information. For these high-dimensional datasets, the goal is to investigate the correlations between large amounts of features (n) often based on a comparably small number of samples (m).

### Efficient computation of correlation matrices

Full correlation matrix computation is the task of calculating the correlations between all feature pairs in a high-dimensional dataset. CorALS employs correlation projections (Fig. 1b) in combination with modern linear algebra routines for low-rank matrix multiplication to calculate complete correlation networks (Fig. 1c, left). The runtime and memory results for this task are shown in Supplementary Data 1 including a comparison with existing software libraries in R. This includes packages such as WGCNA(Weighted Correlation Network Analysis)^27^, Rfast (A collection of Efficient and Extremely Fast R Functions)^28^, coop (Co-Operation: Fast Covariance, Correlation, and Cosine Similarity Operations)^29^ and HiClimR (Hierarchical Climate Regionalization)^30^ with different advantages and disadvantages. For example, WGCNA can handle small amounts of missing values, and HiClimR tries to save memory by calculating only the upper half of the correlation matrix. These methods either use efficient custom C implementations, which are often based on (multi-threaded) nested for-loops (for example, Rfast^28^) or use efficient BLAS (Basic Linear Algebra Subprograms) and LAPACK (Linear Algebra PACKage) matrix multiplication routines with projected vectors similar to CorALS (for example, coop^29^). In addition, we also investigated a selected set of methods from the category of high-performance computing, parallel and distributed frameworks including Deep Graph, Dask and Spark. For a more detailed discussion on these and further alternatives, we refer to Supplementary Section 3. An analysis for varying numbers of features and samples is given in Supplementary Fig. 2. Consistently, CorALS outperforms other approaches and implementations, particularly, the baseline implementations in Julia (statistics.cor), Python (numpy.corrcoef) and R (stats::cor) (Supplementary Data 1). The results for CorALS are based on a Python implementation (single core). Furthermore, the matrix multiplication routines used by CorALS can take advantage of multiple central processing units (here with 64 cores), considerably reducing runtimes (multicore). Note that some baseline implementations (such as Python) also support parallelization. However, their baseline single-core version is already slower than CorALS and thus we skip these experiments, and illustrate only the scaling capabilities of CorALS with increasing computational resources. Other methods from the category of high-performance computing, parallel and distributed frameworks performed slower, did not return results or were not easily available (Supplementary Section 3). Memory requirement differences across all methods are negligible. The cancer and single-cell datasets illustrate how memory requirements can easily exceed the resources of even specialized high-performance computing hardware, when naively calculating full correlation matrices. However, investigating full correlation matrices may not be necessary, as often only the most prominent correlation structures are of interest. This makes focused correlation network construction schemes (for example, based on top-*k* correlations, as introduced in the following sections) useful tools to explore and analyze large-scale correlation structures, while avoiding resource limitations.

### Efficient approximation of large-scale correlation networks

Correlation analysis is often focused on the strongest correlations in a study, that is, by selecting the top-*k* correlations. Straightforward implementations of this approach are based on calculating the overall correlation matrix, and utilizing default sorting algorithms to extract the top-*k* correlations. However, this incurs substantial runtime and memory overhead as illustrated by the baseline implementations in R, Julia and Python (Table 2). To the best of our knowledge, no easy-to-use efficient algorithms exist to calculate only the top-*k* correlations of a given set of features. To address this, for systems with large amounts of memory, CorALS provides a basic algorithm (matrix) that utilizes the previously introduced fast correlation matrix routine (Supplementary Data 1) together with selection algorithms that are able to efficiently partition top-*k* values from the remaining correlations^31^. However, on the one hand, memory requirements quickly exceed available resources (see, for example, memory use in the cancer (0.50) dataset in Table 2), and, on the other hand, the employed partitioning algorithms are not easily parallelizable while consuming the majority of runtime of the top-*k* search (compare matrix in Supplementary Data 1 and matrix in Table 2). To address this, CorALS employs a combination of specific feature vector projections (Fig. 1a), space partitioning techniques and an efficient computation pipeline (Fig. 1b) to approximate the set of strongest correlations in a network (Fig. 1c, middle). The use of efficient space partitioning techniques circumvents calculating the overall correlation matrix, thus avoiding large memory requirements while allowing for substantial runtime improvements (Table 2, index). For more results on runtime, memory and parallelization efficiency for varying numbers of samples and features, also see Supplementary Section 2. The employed approximation scheme trades off resource usage against accuracy. Thus, we provide a theoretical analysis of lower bounds on the amount of potentially found values and the associated sensitivity across various approximation factors (‘Approximate search for global top-k correlations’ in Methods). Note that in practice, the sensitivity associated with specific approximation factors can be much higher then the provided estimates. Consequently, CorALS can even yield perfect results with smaller approximation factors increasing efficiency (Supplementary Section 5). The runtime efficiency of the employed space partitioning techniques grows as the ratio of the number features and samples increases. Thus, particularly on high-dimensional, real-world datasets, CorALS reduces runtimes substantially. In addition, this approach is inherently parallelizable and by employing multiple cores (here, 64), CorALS achieves notable performance gains (for example, from 8 hours to 11 minutes for the cancer (1.00) dataset) and outperforms all baseline for any of the considered datasets by a large margin (parallel). Finally, and importantly, the methods provided by CorALS have a very small memory consumption profile of only a fraction of the baseline implementations. This can be even further reduced depending on the application scenario, for example, by lowering the number of top correlations to extract, introducing explicit correlation thresholds or decreasing the size of the batches in the CorALS computation pipeline (Fig. 1b, middle). Thus, CorALS enables large-scale correlation analyses that are not possible with any of the baseline or basic implementations, even on dedicated high-performance computation hardware (Table 2, cancer (0.5), cancer (1.0) and single cell).

Overall, CorALS allows the calculation of large-scale top-*k* correlation networks on personal computers, enabling accessible workflows that previously required dedicated high-performance infrastructures. For additional runtime, memory and accuracy analyses, see Supplementary Sections 2–5.

### Differential analysis of correlation networks

Differential network analysis^32–34^, and specifically systematically studying the largest differences in correlation networks across more than one condition (or timepoint), can be instrumental to understanding the underlying processes of complex systems^35^. To enable this, CorALS represents features as vectors in a ‘differential space’ (Fig. 1), each of which combines information from two conditions (or timepoints) simultaneously (Fig. 1c). This, allows CorALS to employ an algorithmic approach similar to top-*k* correlation search, enabling efficient top-*k* differential correlation discovery (Fig. 1c, right) with analogous runtime and memory characteristics. Comparable methods such as Differential Gene Correlation Analysis (DGCA) or DiffCor^35,36^ provide approaches for ensuring statistical robustness of their results based on sampling. However, even with sampling disabled, these methods are substantially slower than CorALS. Other approaches such as Discordant and Differential Correlation across Ranked Samples (DCARS)^37,38^, do not allow for top-*k* functionality and thus will quickly run into memory issues. Thus, CorALS allows for a much more efficient discovery of top-*k* correlation discovery. For a more in-depth discussion, we refer to Supplementary Section 3.3. To ensure robustness, either CorALS can be used as an efficient candidate selection step, which can then potentially be tested with the methods mentioned above, or similar sampling techniques can be implemented in CorALS. We apply such a sampling approach in ‘Large-scale multiomics correlation analysis across pregnancy’, where we take advantage of the efficient runtime characteristics of CorALS to account for spurious correlations by employing a corresponding sampling-based strategy.

### Explicit visualization of correlation structure

**Fig. 2 |:**
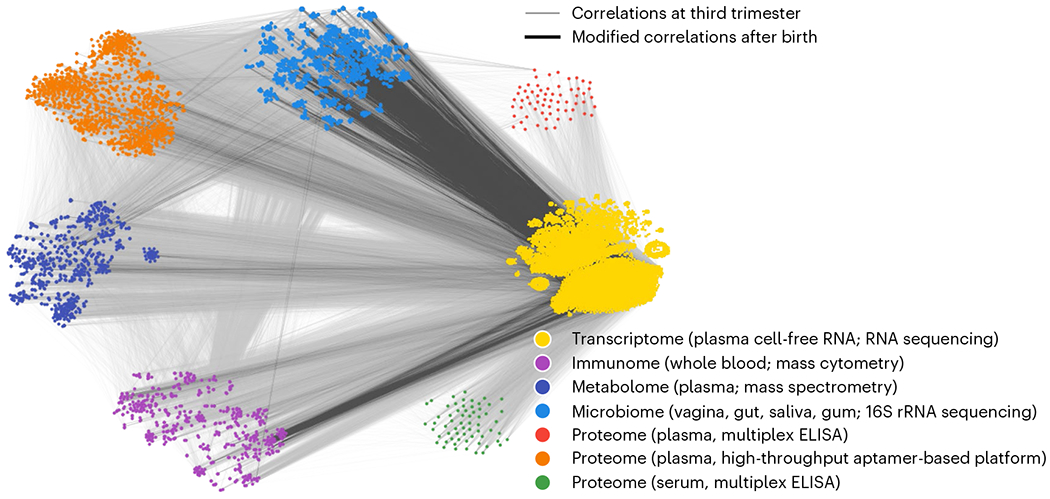
Dynamic changes in multiomic measurements before and after birth. Nodes represent individual features arranged with CorALS’s correlation-based t-SNE for each omic. Omics were measured using different technologies (enzyme-linked immunosorbent assay, ELISA), and are visualized separately from each other. Light-gray edges correspond to the top-10% correlations in the third trimester. Of those top correlations, the dark edges in the foreground correspond to the 0.01% of correlations that change the most from third trimester to postpartum. Coordinated differences are apparent, for example, related to the correlation of pP38 phosphorylation in various immune-cell subtypes and specific gene transcripts detected among cell-free RNA.

**Fig. 3 |:**
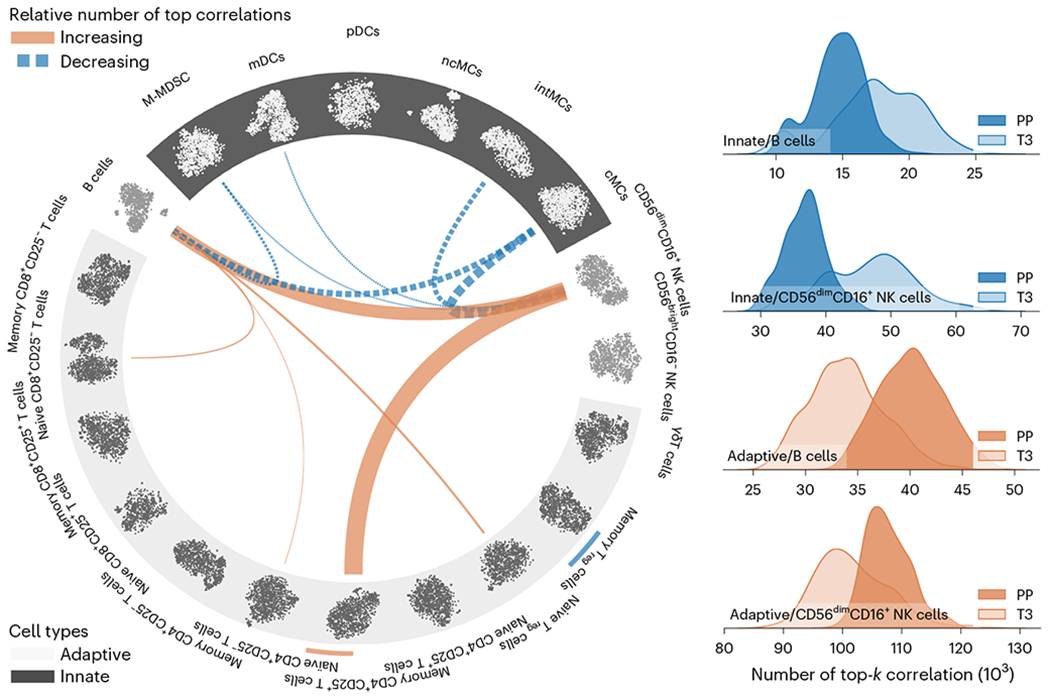
Concerted immune regulation at the single-cell level during pregnancy. Left: pairs of cell types with substantially different (very large effect size, t>0.622) relative numbers of top- k(k=0.01%) correlations of individual cells between the third trimester (T3) and postpartum (PP) based on the cells’ functional characteristics. For cell type abbreviations, see Supplementary Table 3. The thickness of the connecting edges represent the corresponding effect size (Cliff’s δ) across samples. Only very large effect sizes are visualized (threshold =0.622 ). The blue and orange colors of the edges signifies a relative decrease or increase, respectively, in cell correlations from the third trimester to postpartum. The scatter plots along the circle show single cells from each cell type visualized using CorALS’s correlation-based t-SNE (innate, dark background; light cells, adaptive; light background, dark cells). Right: the accumulated top- k correlation shifts between the innate and adaptive immune cells, and B cells and CD56 ${}^{dim}CD16^{+}$NK cells, respectively, shown by density plots for the number of top- k correlations across samples. Overall, this visualization illustrates the dynamically changing overlap of functional characteristics of B cells and CD56 ${}^{dim}$ CD16 ${}^{+}$NK cells with the functional characteristics observed in the total pool of innate or adaptive immune-cell populations.

Feature embeddings are an essential tool to represent and analyze features in low-dimensional spaces. For example, Fig. 2 shows features visualized using *t*-distributed stochastic neighbor embeddings (t-SNE). However, t-SNE is generally based on Euclidean distances and thus does not directly represent the correlation structure of features. Although some t-SNE implementations support custom correlation-based distance information, this is often inefficient owing to algorithmic overhead. To address this, CorALS uses correlation projections (Fig. 1a, middle) to exploit the direct relationship between correlation and Euclidean distance (Supplementary Section 6). This allows to employ any existing distance-based method for embedding features without adding substantial computational overhead. All feature and cell visualizations throughout this paper are based on this approach (for example, Figs. 1–3).

### Large-scale multiomics correlation analysis across pregnancy

Understanding maternal biological changes during and immediately after pregnancy is a fundamental step to improving diagnostic and therapeutic strategies in peripartum management, to prevent critical conditions that extend well into the child’s adulthood (for example, preterm birth, the single largest cause of death in children under 5 years of age). Despite this, previous studies have not investigated possible changes in the cross-talk across various biological modalities^6,39^. To demonstrate CorALS’s utility in high-dimensional multiomics studies, we analyzed a dataset containing third trimester and postpartum measurements of biospecimens from 17 healthy pregnant women^6^. Each sample in the corresponding data contains more than 60,000 synchronized measurements from seven different omics (Fig. 2) from which we selected ~41,000 by filtering features with missing or constant values. Details on assays and the measured biomarkers can be found in ‘Datasets’ in Methods.

We used CorALS to calculate the top-10% Spearman correlations between all feature pairs for the third trimester. Furthermore, we extracted the top-0.1% strongest differential correlations in contrast to postpartum by employing CorALS’s corresponding implementation. To focus the results on strong signals, we selected feature pairs passing a correlation threshold of 0.8 in the third trimester for further analysis. For visualization (Fig. 2), we utilized CorALS’s correlation-based feature embeddings based on t-SNE^40^ for each individual omic.

The visualization reveals various prominent changes in correlation structure between the different omics from the third trimester to postpartum. In particular, the correlation changes between transcriptome and microbiome, as well as between the transcriptome (cell-free RNA) and immunome (including phenotypical, and the functional markers measured by mass cytometry or cytometry by time of flight, are prominent. These correlations appear in the third trimester but vanish postpartum (edges marked in dark gray). Refer to the Supplementary Section 8 for details and an expanded biological analysis.

Overall, while establishing causal links requires careful follow-up studies and biological validations, the results outlined in Supplementary Section 8 are a powerful example that illustrates how the efficient analysis of large-scale correlation networks as enabled by CorALS can drive the generation of biological hypotheses.

### Correlated functional changes across immune cells

While recent advances in single-cell technologies have enabled the production of large immunological datasets, data analysis approaches for single-cell data have remained limited to traditional analysis of changes in the frequency and signaling pathways of cell types. In this example, we demonstrate that CorALS allows to derive a complementary perspective on the dynamic coordination of functional characteristics across several immune-system components on the single-cell level. We analyzed a dataset of more than 24 million cells from 17 participants tracking the immune system through pregnancy using mass cytometry^1^. Notably, this dataset contains simultaneous measurements of both phenotypic markers as well as intracellular proteins, the latter serving as markers for endogenous signaling responsiveness of single cells. The phenotypic markers were used to identify various cell populations via manual gating^1^, and CorALS was used to study shifts in cell similarities across the signaling pathways of various cell types using the available ten functional markers (Fig. 3) based on Spearman correlation. To increase the robustness of the dynamic changes identified, this analysis requires repeated sampling and top-*k* correlation calculations across millions of individual cells, making CorALS an essential component of the analytical pipeline by substantially reducing runtime and memory requirements (processing a single sample corresponds to the single-cell experiment in Table 2). Refer to the Supplementary Section 9 for details and an expanded biological analysis.

Figure 3 shows a summary of this analysis and visualizes the amount and direction of change in the relative number of functional cell correlations attributed individual cell type pairs within the top-*k* functional cell correlations between the third trimester and postpartum. These changes mostly revolve around B cells and CD56^dim^CD16^+^ natural killer (NK) cells. While a detailed analysis may be of interest, we focus on these changes as an example to illustrate the complementary perspectives enabled by CorALS. In general, from the third trimester to postpartum, B cells and CD56^dim^CD16^+^ NK cells show a higher degree of similarity in terms of signaling response signatures postpartum (orange edges) to cell types of the adaptive immune system (light background). At the same time, they share less similarity (blue edges) in their intracellular signaling response signature with the cells of the innate immune system (dark background). We further visualize this trend through density plots in Fig. 3, directly comparing the number of top-*k* correlations of B cells and CD56^dim^CD16^+^ NK cells, respectively, with the total pool of innate or adaptive immune-cell subsets in the third trimester versus postpartum. This analysis provides a complementary perspective on the coordination of single-cell systems during pregnancy, and suggests that B cells and CD56^dim^CD16^+^ NK cells acquire innate-like functional characteristics in the third trimester, and that, postpartum, these two cell types and various T-cell subsets shift functionally to resemble each other.

On the basis of these conjectures, and given further datasets for validation, the changes observed in Fig. 3 may guide further research on the role of B cells and CD56^dim^CD16^+^ NK cells and the phenotypes they acquire over the course of pregnancy. This serves as a practical example on how CorALS can enable complementary perspectives on many different domains, including the coordination of single-cell systems, by enabling the efficient implementation and application of large-scale correlation analysis.

## Discussion

Modern biological profiling techniques will enable the collection of datasets with increasingly high dimensions and sample sizes. Therefore, the consistent analysis of evolving datasets will require continuous improvements. We can further advance CorALS with advanced indexing and sorting algorithms, on-disk sorting algorithms, or employing distributed computing environments. The computational pipeline of CorALS is designed to support such extensions.

For example, the current version of CorALS is optimized for high-dimensional datasets with small sample sizes. However, as sample sizes increase, the efficiency of the employed indexing structure can deteriorate. Alternatively, approximate indexing structures increase runtime in exchange for sensitivity. Also, approaches based on a batched computation of partial correlation matrices combined with thresholding may be an alternative (see ‘Feature projections’ in Methods for details). However, the latter approach will require careful balance between the number of batches, the number of concurrent tasks, threshold size, memory availability and runtime, as a threshold does not provide memory guarantees. To tackle this, various methods to cache data outside of the main memory can be employed. A principled approach to this are distributed frameworks, for example, based on MapReduce^41^. CorALS already supports such distributed computation on various backends. We provide a Jupyter notebook that exemplifies running CorALS on a Spark cluster^42^. However, while the implementation of CorALS already contains many of the previously mentioned extensions, a detailed comparison and analysis is beyond the scope of this work. For further practical consideration, also see ‘Practical considerations’ in Methods.

Also, the CorALS implementation provides tools to derive *P* values to gauge the significance of the measured correlations (‘P-value calculation and multiple testing correction’ in Methods), and supports the non-parametric Spearman correlation (‘Correlation coefficient classes’ in Methods) to account for outliers or certain error types^8,43^. However, *P* values and the Spearman coefficient do not generally address challenges such as data errors and noisy data. To tackle this issue, correlation measures are often calculated based on computationally expensive techniques, for example, based on bootstrapping^43^, making their application in high-dimensional data impractical. In this context, CorALS can be used either to efficiently sample correlations using full correlation matrix calculation or to first select top-*k* correlations for which robust methods can then be applied selectively. Similarly, CorALS does not account for confounding or causation. However, more advanced approaches to account for these effects, such as partial correlation or Bayesian networks^44,45^, are often restricted to small datasets and do not scale for high-dimensional data. In this context, CorALS can be used to effectively suggest highly correlated components of the data for further investigation with such methods. Thus, overall, investigating correlation networks can be broadly applied to gain insight into the underlying functional structures, which then may provide input for downstream analysis and also for more advanced methods such as graph neural networks^11,12^.

Finally, as the number of features increases with advancing technologies, it may be necessary to introduce more sophisticated methods that find correlated compounds, for example, based on existing domain knowledge, rather than individual correlations, for which CorALS can lay the computational foundation.

Overall, owing to its wide range and scope, we anticipate CorALS to be a catalyst that will be adopted to enable a multitude of downstream applications of large-scale correlation networks. For example, in ‘Correlated functional changes across immune cells’, the efficiency characteristics of CorALS’s top correlation network estimation allow to derive an innovative sampling-based approach to analyze the interaction of hundreds of thousands of cells simultaneously. In future work, CorALS may also support advanced tensor and network analysis or deep learning and graph neural network modeling (for example, for gene-interaction graphs and cell-to-cell relationships^11,12^). Thus, it will lay the analytical foundations and provide computational tools to unravel the intricate interactions of biological systems as developing computational approaches are able to analyze increasingly complex network structures.

## Methods

### Derivation of efficient feature representations by CorALS

The different components of CorALS rely on transforming features into specific vector representations that connect the scalar product of these vectors to efficient correlation computations. In the following, we outline the derivation of these transformations for correlation projections (used for efficient correlation matrix calculation, top correlation network approximation and correlation embeddings) as well as differential projections (used for top differential correlation search), respectively. It is noted that the following feature representations are derived for the Pearson correlation coefficient; however, without loss of generality, these derivations hold for Spearman’s rank correlation coefficient by replacing individual feature values with ranks per feature. This is supported by CorALS’s implementation.

#### Correlation projections.

By transforming feature representations appropriately, correlation computation can be formulated as a scalar product of two pre-processed vectors^46^. We refer to this pre-processing step as correlation projection. In particular, the Pearson correlation cor(x,y) between two features x and y with respective sample vectors $x=\left(x_{1},\ldots ,x_{m}\right)$ and $y=\left(y_{1},\ldots ,y_{m}\right)$, can be rewritten as follows:

(1) $$\text{cor}(x,y)=\frac{\sum_{i=1}^{m}\left(x_{i}-\mu_{x}\right)\left(y_{i}-\mu_{\text{y}}\right)}{\sqrt{\sum_{j=1}^{m}{\left(x_{j}-\mu_{x}\right)}^{2}\sum_{j=1}^{m}{\left(y_{j}-\mu_{y}\right)}^{2}}}\;=\sum_{i=1}^{m}\frac{\left(x_{i}-\mu_{x}\right)}{\sqrt{\sum_{j=1}^{m}{\left(x_{j}-\mu_{x}\right)}^{2}}}\frac{\left(y_{i}-\mu_{y}\right)}{\sqrt{\sum_{j=1}^{m}{\left(y_{j}-\mu_{y}\right)}^{2}}}\;=\langle \frac{x-\mu_{x}}{\sqrt{\sum_{i=1}^{m}{\left(x_{i}-\mu_{x}\right)}^{2}}},\frac{y-\mu_{y}}{\sqrt{\sum_{i=1}^{m}{\left(x_{i}-\mu_{\text{y}}\right)}^{2}}}\rangle \;=\langle \hat{x},\hat{y}\rangle \;\text{with}\;\hat{z}=\frac{z-\mu_{z}}{\left\|z-\mu_{z}\right\|}$$

where $\mu_{z}$ is the mean of vector z. Thus, the $\overset{ˆ}{•}$ operator corresponds to the correlation projection that allows the transformation of the original sample vectors so that their scalar product is equal to their correlation. CorALS exploits this vector representation to formulate correlation matrix computation as an efficient matrix product.

This transformation allows to derive a direct relationship between the correlation cor(x,y) of any two vectors and the Euclidean distance $d_{e}(\overset{ˆ}{x},\overset{ˆ}{y})$ of their correlation projections^46^. In particular, cor(x,y) and $-d_{e}(\overset{ˆ}{x},\overset{ˆ}{y})$ are order-equivalent and it holds that:

(2) $$\text{cor}(x,y)=1-\frac{d_{\text{e}}{\left(\hat{\text{x}},\hat{\text{y}}\right)}^{2}}{2}$$

CorALS exploits this relationship between correlation and Euclidean distance, for example, in top correlation approximation and correlation-based embeddings. For more details and corresponding proofs, see Supplementary Section 6.1.

#### Differential projections.

CorALS further introduces a dual feature representation in a differential space that allows to calculate correlation differences across two conditions or timepoints using a single scalar product. In particular, for two features x and y, let $x_{1}=\left(x_{1,1},\ldots ,x_{1,m_{1}}\right)$ and $y_{1}=\left(y_{1,1},\ldots ,y_{1,m_{1}}\right)$ denote respective sample vectors in the first condition/timepoint and $x_{2}=\left(x_{2,1},\ldots ,x_{2,m_{2}}\right)$ and $y_{2}=\left(y_{2,1},\ldots ,y_{2,m_{2}}\right)$ in the second condition/timepoint. Then, the goal is to find vector transformations $\delta \left(x_{1},x_{2}\right),\kappa \left(y_{1},y_{2}\right)$ that represent information form both conditions/timepoints simultaneously so that

(3) $$\text{cor}\left(x_{1},y_{1}\right)-\text{cor}\left(x_{2},y_{2}\right)=\langle \delta \left(x_{1},x_{2}\right),\kappa \left(y_{1},y_{2}\right)\rangle$$

Given the correlation projection $\overset{ˆ}{•}$ from ‘Correlation projections’, the following definitions for δ and κ provide such a dual vector representation.


(4) $$\delta :{\mathbb{R}}^{m_{1}}\times {\mathbb{R}}^{m_{2}}\to {\mathbb{R}}^{m_{1}+m_{2}}\;z_{1},z_{2}\mapsto \left(\begin{array}{c} {\hat{z}}_{1}\\ {\hat{z}}_{2} \end{array}\right)\;\kappa :{\mathbb{R}}^{m_{1}}\times {\mathbb{R}}^{m_{2}}\to {\mathbb{R}}^{m_{1}+m_{2}}\;z_{1},z_{2}\mapsto \left(\begin{array}{c} {\hat{z}}_{1}\\ -{\hat{z}}_{2} \end{array}\right)$$

We call the vector space containing the codomain of these functions differential space.

Similar to the connection of Euclidean distance and basic correlation (see above), the dual feature representations in the differential space exhibit a connection between Euclidean distance and correlation difference across conditions or timepoints. In particular, for two features x and y with sample vectors $x_{1},x_{2}$ and $y_{1},y_{2}$ across two conditions or timepoints, $cor\left(x_{1},y_{1}\right)-cor\left(x_{2},y_{2}\right)$ and $-d_{e}\left(\delta \left(x_{1},x_{2}\right),\kappa \left(y_{1},y_{2}\right)\right)$ are order-equivalent and it holds that:

(5) $$\text{cor}\left(x_{1},y_{1}\right)-\text{cor}\left(x_{2},y_{2}\right)=2-\frac{d_{\text{e}}{\left(\delta \left(x_{1},x_{2}\right),\kappa \left(y_{1},y_{2}\right)\right)}^{2}}{2}$$

Thus, analogously to correlation projections, CorALS exploits this order equivalence of Euclidean distance and correlation differences for top differential correlation approximation. For more details and corresponding proofs, see Supplementary Section 6.2.

### Efficient calculation of full correlation matrices

Efficiently calculating full correlation matrices is achieved by recognizing that the inner product formulation in equation (1) allows to condense the correlation calculation between all possible feature pairs in a dataset to a single matrix product ${\overset{ˆ}{X}}^{⊤}\overset{ˆ}{X}$. Here, $\overset{ˆ}{X}\in R^{m\times n}$ is the sample-feature matrix representing the corresponding dataset with m samples and n features where each column corresponds to the correlation projected sample vector of each feature, respectively (see ‘Correlation projections’). This approach can be directly formulated in any recent programming language without requiring additional software packages, and is able to take advantage of built-in efficient linear algebra routines such as BLAS and LAPACK^47,48^, which inherently support parallelization as showcased in Supplementary Data 1 and Supplementary Section 3. This approach outperforms many other implementations employing similar concepts as demonstrated in Supplementary Table 2.

### Efficient approximation of correlation networks

#### Top correlation computation as a query search problem.

By default correlation networks are fully connected. However, often it is more valuable to study only the most interesting interactions, that is, the strongest correlations. For this, it is common to either define a fixed threshold or concentrate the analysis on the top-*k* correlations. A straightforward approach to achieve this is to calculate the full correlation network and then keep only those correlations that are sufficiently strong according to either criterion. However, for high-dimensional data, calculating the full correlation matrix between features is often not feasible owing to memory restrictions, and in the top-*k* case, the subsequent sorting operation has more than cubic complexity with the number of features $n\left(𝒪\left(n^{2}\;log\;n\right)\right)$. And even when using partial sorting techniques based on selection algorithms for top-*k* search, this may result in impractical runtimes $\left(𝒪\left(n^{2}+k\;\log\;k\right)\right)$^31,49^.

To address this, we fist observe that owing to the symmetry property of correlation measures, a single feature can never be strongly correlated to all other features (except in cases where all features highly correlated). Thus, we assume that the top global correlations can be approximated by finding and merging the top correlations locally, for example, for each feature separately, given an appropriate local margin (coined approximation factor as introduced below). This allows CorALS to reinterpret the task of top correlation computation as a query search problem^50^ where an indexed set of elements is efficiently queried based on a set of query vectors and a given distance measure. In particular, CorALS constructs an efficient index structure $T_{X}$ over a set of features X and then interprets another (often the same) set of features as queries Y to find the top correlated feature pairs. This approach prevents the construction of the complete correlation matrix and the corresponding implementation is inherently parallelizable, resulting in substantially reduced runtimes and memory requirements.

In the following, we describe the individual steps to enable this approach. This includes (1) the construction of an optimized indexing and query method that circumvents limitations of the previously derived relation between Euclidean distance and correlation (‘Joint ball trees for local top correlation discovery’), (2) the description of an approximation scheme to generalize single-query-based search to return global top-*k* correlations (‘Approximate search for global top-*k* correlations’), and (3) a discussion on the implementation of threshold-based search (‘Threshold-based correlation filtering’).

#### Joint ball trees for local top correlation discovery.

While in principle, any metric-based k-nearest-neighbor algorithm can be used for CorALS, we focus on space partitioning algorithms that allow for efficient top-*k* as well as threshold-based queries in high-dimensional settings. Ball trees (or metric trees) in particular automatically adjust their structure to the represented data, provide good average-case performance and can cope well with high-dimensional entities^50,51^. While such indexing structures are mostly optimized for metrics such as the Euclidean distance, CorALS takes advantage of the correlation projection introduced in ‘Correlation projections’ and its properties (see ‘Correlation projections’) to enable top correlation and differential correlation search.

In particular, CorALS first represents each feature as a correlation vector by applying the correlation projection introduced in ‘Correlation projections’ to their respective sample vectors. These correlation vectors X are then indexed using ball tree space partitioning resulting in index $T_{X}$. On the basis of the relation between Euclidean distance and correlation derived in ‘Correlation projections’, this index allows to search for top-*k* positively correlated features search$\left(T_{X},y,k\right)$ based on a given query feature y∈Y. It also allows to search for a set of features search$\left(T_{X},y,t\right)$ passing a positive correlation threshold t with respect to the query feature y.

Note that this set-up has two specific limitations that we address in the following. First, ball trees generally only support to search for top correlations relative to a single reference feature y. The algorithm to generalize this to a set of features will be described in ‘Approximate search for global top-k correlations’ and ‘Threshold-based correlation filtering’. Second, by default, only feature pairs with positive correlations are returned because only positive correlations correspond to small Euclidean distances while negative correlations will result in large distances (see equation (2) and the corollary in Supplementary Section 6.1).

To address the latter, CorALS takes advantage of the fact that correlation (as well as the scalar product) is associative with respect to scalar multiplication. In particular, changing the sign of a sample vector also changes the sign of the correlation:

(6) cor(−x,y)=−cor(x,y)=cor(x,−y)

Now, without loss of generality, we focus on top-*k* search in the following derivation. Assuming that at least k features with positive correlations to a query feature y exist in X, then all correlations returned by search$\left(T_{x},y,k\right)$ are positive. Similarly, assuming that at least k negative correlations exist, switching the sign of all features in the dictionary X, that is, search$\left(T_{-x},y,k\right)$, or switching the sign of the query, that is, search$\left(T_{x},-y,k\right)$, allows to also extract the strongest negative correlations (see equation (6)). Thus, a simple solution to find those features with the top positive and negative correlations is to run the search twice, once to extract positive and once to extract negative correlations, followed by a merging step.

However, for top-*k* search, this merging step, involves returning the top-*k* correlations twice, resulting in a sorting step that orders 2k elements, which can double memory requirements. This can be prevented by building the ball tree based on positive and negative dictionary features simultaneously, that is, search$\left(T_{-xux},y,k\right)$. This search only returns k elements, and thus can reduce runtime and memory requirements. See Supplementary Table 1 for a comparison of top-0.1% search on real-world datasets (Table 1). The corresponding experiments are based on the CorALS’s Python implementation and were repeated ten times; reported medians had no substantial fluctuations between runs. While the runtime improvements are marginal, the memory consumption can be reduced by half. Also note that for multiple queries, ball trees support to pre-process the set of queries resulting in a dual-tree approach^52^ for speeding up the search. Supplementary Table 1 also demonstrates the effectiveness of this approach. For the final implementation of CorALS, we jointly build the ball tree structure on negative and positive features and employ the dualtree search whenever provided by the underlying software library.

#### Approximate search for global top-*k* correlations.

Focusing on the top-*k* correlations can be an effective way to construct interpretable visualizations of correlation matrices without having to explicitly specify a threshold. For this, k is often large, defined either as a multiple of the number of features (for example, 100n,1,000n), or as a percentage (say 0.1% of all correlations $∼\left\lceil n^{2*}0.001\right\rceil$). However, the ball tree algorithm (see ‘Joint ball trees for local top correlation discovery’) returns only the top correlations for each feature rather than the overall top-*k* correlations between all features. To address this, CorALS employs an approximation scheme.

In particular, for each query feature y∈Y, CorALS heuristically sets the number of $k^{\prime }$ top correlated features to retrieve and then merges the results to approximate the global set of top-*k* features. Selecting $k^{\prime }$ presents a trade-off. On the one hand, if $k^{\prime }$ is greater than or equal to the number of features n, all feature pairs will be considered, thus allowing for an exact determination of the top-*k* features but no gain in runtime. On the other hand, if $k^{\prime }<n$, then there is no guarantee that the exact top-*k* features are retrieved; however, the runtime can be substantially improved as only a subset of candidates is returned and processed. To address this, CorALS uniformly draws top correlation candidates across all query features with a sufficient margin that accounts for biases in the correlation structure. That is, we chose $k^{\prime }$ to be dependent on k with $k^{\prime }=a\left\lceil \frac{k}{n}\right\rceil$ as a middle ground between drawing the exact number of required candidates from each query $k^{\prime }=\left\lceil \frac{k}{n}\right\rceil$ and considering all candidates from each query $k^{\prime }=n$. Here *a* is called the approximation factor and regulates how many correlations are inspected per feature. The approximation factor can be selected so that CorALS returns results up to a specific sensitivity s. In particular, for a desired sensitivity up to s≤0.75, the approximation factor can be chosen based on $a=s\frac{n}{\sqrt{k}}$; and for a desired sensitivity s≥0.75, the approximation factor can be chosen based on $a=\frac{sn}{2\sqrt{k}\sqrt{1-s}}$. When formulating k in terms of the overall number of correlations $n^{2}$, that is, $k=rn^{2}$, for a sensitivity of s≤0.75, the approximation factor can be calculated via $a=\frac{s}{\sqrt{r}}$, and for s≥0.75 it can be calculated via $a=\frac{s}{2\sqrt{r}\sqrt{1-s}}$. However, in practice the number of missed correlations can be substantially smaller as correlations are usually not distributed according to the the worst case (Supplementary Figure 5). The derivation of sensitivity estimates as well as a study of the effects of a itself can be found in Supplementary Section 5. Supplementary Algorithm 1 summarizes the overall approach.

#### Threshold-based correlation filtering.

To calculate all correlations greater than a threshold t, for each feature y∈Y, we can also employ the ball tree data structure (see ‘Joint ball trees for local top correlation discovery’) by issuing radius queries. For this, the correlation threshold needs to be converted into an Euclidean radius using equation (2). Thus, for each query feature, the respective query returns all indexed features with correlations greater than the respective correlation threshold. The results of each query are then merged to retrieve the final list of the filtered feature pairs. This approach is more memory efficient than calculating correlations for all possible feature pairs, for example, using the methodology introduced in ‘Efficient calculation of full correlation matrices’. However, it can also result in substantially increased runtimes compared with calculating the complete correlation matrix. The corresponding algorithm is implemented analogously to the top-*k* search in Supplementary Algorithm 1 but replaces k with a correlation threshold that is converted into a corresponding Euclidean radius via equation (2) to be used by the ball tree index structure.

### Top correlation difference search

To efficiently calculate the top differences in correlation between pairs of features across more than one timepoint or condition, the naive implementation involves calculating the full correlation matrices for two conditions or timepoints, subtracting them and then extracting the top differences, for example, through thresholding or by identifying the top-*k* candidates. As previously shown for top-*k* correlation search, this is runtime and memory extensive if implemented naively and thus can easily exceed computational resources (Table 2).

To address this, CorALS builds on the dual feature representation introduced in ‘Differential projections’. In particular, it exploits the connection of correlation difference and Euclidean distance between the dual representation of features in differential space and then applies the same query search approach as for top correlation search (see ‘Efficient approximation of correlation networks’).

Thus, this first requires representing all features x∈X as their dual representations δ(x)∈δ(X) and κ(x)∈κ(X). Then, analogously to ‘Joint ball trees for local top correlation discovery’, a combined ball tree $T_{\delta (X)\cup -\delta (X)}$ is constructed to cover negative as well as positive differences. This ball tree can then be used to query the top-*k* (or thresholded) correlation differences search$\left(T_{\delta (X)\cup -\delta (X)},y,k\right)$ by querying with the feature representations κ(x)∈κ(X). This already includes positive and negative correlation differences as we index positive and negative projections δ(X)∪−δ(X), while indexing only δ(X) would solely return the top positive correlation differences (see equation (2) and the corollary in Supplementary Section 6.2). After the construction of $T_{\delta (X)\cup -\delta (X)}$, the same approximation approach as laid out in ‘Approximate search for global top-k correlations’ and ‘Threshold-based correlation filtering’ is employed to query the top correlation differences across all query features κ(X).

### Correlation embeddings

t-SNE^40^ was used to embed high-dimensional data points into low-dimensional spaces, for example, for visualization. In this work, we employ t-SNE to embed features based on their correlation structure across samples. However, t-SNE is based on Euclidean distance and thus does not directly represent the correlation structure of features.

In particular, t-SNE reduces the dimensionality of data by minimizing the Kullback–Leibler divergence between a probability distribution, P, in the high-dimensional space and a probability distribution, Q, in the low-dimensional space^40^:

(7) $$C=KL(P∥Q)=\sum_{i}\sum_{j}p_{ij}\text{log}\frac{p_{ij}}{q_{ij}}$$

where the probabilities $p_{ij}$ and $q_{ij}$ represent probabilities for features j to belong to the neighborhood of feature i based on Euclidean distance in the corresponding space:

(8) $$p_{ij}=\frac{\text{exp}\left(-{\left\|z_{i}-z_{j}\right\|}^{2}\right)/2\sigma^{2}}{\sum_{k\neq l}\text{exp}\left(-\left\|z_{k}-z_{l}\right\|\right)/2\sigma^{2}}\;q_{ij}=\frac{{\left(1+{\left\|{\tilde{z}}_{i}-{\tilde{z}}_{j}\right\|}^{2}\right)}^{-1}}{\sum_{k\neq l}{\left(1+{\left\|{\tilde{z}}_{k}-{\tilde{z}}_{l}\right\|}^{2}\right)}^{-1}}$$

with ${∥z_{i}-z_{j}∥}^{2}$ and ${∥{\tilde{z}}_{i}-{\tilde{z}}_{j}∥}^{2}$ representing pairwise Euclidean distances between features i and j for high-dimensional z and low-dimensional feature representations $\tilde{z}$, respectively.

Now, by projecting features onto correlation vectors, CorALS establishes an order equivalence between Euclidean distance and correlation as introduced in ‘Correlation projections’. This allows to directly employ distance-based embeddings methods such as t-SNE on the projected features without adding substantial computational overhead or requiring implementations that support customized distance information. A performance example is given in Supplementary Section 7.

### Correlation coefficient classes

The underlying computation of CorALS is based on the Pearson correlation coefficient as discussed in the previous sections. On this basis, CorALS also supports any class of correlation coefficients that can be reduced to the Pearson calculation scheme. In particular, Spearman correlation can be calculated using the Pearson formula by replacing individual feature values with feature-local ranks, which may help to account for outliers or certain error types^8,43^. CorALS provides the corresponding capabilities to switch between Pearson and Spearman. Similarly, the Phi coefficient for binary variables can be calculated using the Pearson formula^53^. Finally, other correlation coefficient classes may be supported by future versions of CorALS by finding a mapping between the corresponding coefficient and Euclidean distance as derived in the previous section for the Pearson correlation coefficient.

### *P*-value calculation and multiple testing correction

*P* values for Pearson correlation coefficients r, can be derived from the correlation coefficient together with the number of samples n. That is, first the t-statistic can be derived using $t=r\frac{\sqrt{n-2}}{\sqrt{1-r^{2}}}$. Then, the P value can be calculated by examining the cumulative t-distribution function p:P=2⋅p(T>t) where T follows a *t*-distribution function with N−2 degrees of freedom. This approach is implemented in CorALS as derive_pvalues and can be applied as a post-processing step.

Note that owing to the large amount of correlations calculated, multiple test correction is necessary when working with P values. The most straightforward approach is to control for family-wise error rate using Bonferroni correction, which multiplies the corresponding *P* values by the number of compared correlation coefficients $\frac{n^{2}-n}{2}$. Other approaches such as the false discovery controlling procedure Benjamini–Hochberg generally require the full *P* value distribution, which is not available when applying top-*k* correlation discovery. In these cases, padding the calculated *P* values with 1s for unknown *P* values can provide an upper bound for adjusted *P* values. However, this generally requires instantiating the full number of *P* values, which causes memory issues like in the full correlation matrix case Supplementary Table 1 . To address this we provide a truncated version of the Benjamini–Hochberg procedure that avoids this issue.

The Benjamini–Hochberg (BH) procedure yields adjusted *P* values^54^ through

(9) $$P_{\left(i\right)}^{\text{BH}}=\text{min}\left\{{\text{min}}_{j\geq i}\left\{\frac{m\cdot P_{j}}{j}\right\},1\right\}$$

with $P_{\left(i\right)}^{BH}$ representing the BH corrected *P* value at rank (i) for ascendingly ranked P values, m being the number of overall *P* values, for example, $m=\frac{\left(n^{2}-n\right)}{2}$, and j represents the rank of the *P* value $P_{j}$. On the basis of this formula, a truncated upper-bound version of BH calculates the adjusted *P* values for all top-*k P* values. Then a upper-bound adjusted value is calculated by $u=\frac{m\cdot 1}{k+1}$. If $P_{k}>u$, then all adjusted P values P with $P=P_{k}$ are replaced by u. This yields a minimally invasive truncated BH procedure for adjusted P values without instantiating the full distribution of P values. The approach is implemented in CorALS as multiple_test_correction and can be applied as a post-processing step.

### Extensible framework for large-scale correlation analysis

The computational framework of CorALS is based on three steps (Fig.1b): a *f*eature projection step, a dynamic batching step and a reduction step. As such, the general structure is compatible with the the big data computation model MapReduce^41^.

The feature projection step (Fig. 1b, left) allows for preparing the data so that it can be split and processed independently in an efficient manner. In this paper, we specifically focus deriving an indexing structure based on space partitioning that allows for efficiently querying top-*k* correlations.

The dynamic batching step (Fig. 1b, middle) then splits the data matrix into multiple batches. The prepared data (and indexing structures) are then used to locally extract the relevant values in each batch independent of the other batches. Batches can be processed sequentially, in parallel or even in a distributed manner. Thus, the smaller the batches and the smaller the number of batches that run simultaneously, the less memory is required. This fine-grained control over batches introduces an effective mechanism to manage and trade-off memory requirements and runtime based on the available resources. Furthermore, batches may store their results on disk rather than in-memory, further reducing memory requirements. In this paper, for each batch of features, we focus on utilizing the previously mentioned indexing structure to extract the local top-*k* correlations in line with the corresponding approximation factor (see ‘Approximate search for global top-k correlations’). We also provide a thresholding feature that can reduce memory requirements of the batch results.

Finally, the batch results are reduced into the final result by merging batches. Dependent on the batch implementation and the local results, this can be done directly in memory for the fastest runtimes, sequentially by merging one batch result at a time or even mostly on disk, which can be used to further reduce memory requirements in favor of computation time. In the implementation of the final join analyzed in this paper, the results from the batches consist of individual correlations, which are merged, partitioned and then sorted to return the final top-*k* values.

#### Feature projections.

Note that the implementation provided by CorALS is highly extensible and nearly all aspects can be replaced by custom implementations to optimize for particular application scenarios. For example, during the feature projection step, the index structures employed in the current implementation are based on ball trees, which optimizes for high-dimensional datasets with small samples sizes by employing correlation and differential spaces (Fig. 1a). However, this index structure can easily be replaced by implementations with different computational characteristics. For example, it may make sense to consider approximate nearest-neighbor methods^55^ to replace the current index, which may potentially reduce runtimes for a cut in sensitivity. Similarly, particularly for larger sample sizes, instead of using indexing structures, it may be advantageous to directly calculate correlations for smaller batches via the efficient matrix multiplication scheme introduced in ‘Efficient calculation of full correlation matrices’. While this direct calculation and partitioning of correlations increases time complexity from 𝒪(nlogn) to $𝒪\left(n^{2}\right)$, this may be faster than the currently employed ball tree indexing structure as the corresponding search performance of 𝒪(logn) may deteriorate to 𝒪(n) with increasing dimensionality (in our case sample size). Here it is important to appropriately select the number of simultaneous batches to limit the memory requirements of this approach (for example, if only one batch is used, the complete correlation matrix will be instantiated). A corresponding implementation is provided by CorALS. A detailed comparison with in-depth parameter optimization and the corresponding relation to more efficient approximate nearest-neighbor schemes is left for future studies.

#### Distributed computation.

The methods in this paper are focused on in-memory computations. However, as mentioned earlier, the computational framework of CorALS allows for sequential computation of batch results which can be cached on disk, circumventing potential memory limitations and allowing for calculating correlations for massive datasets. Furthermore, CorALS also supports distributed computation of correlation and differential matrices through the joblib backend (https://github.com/joblib/joblib). This directly enables Spark (https://github.com/joblib/joblib-spark), Dask (https://ml.dask.org/joblib.html) or Ray (https://docs.ray.io/en/latest/joblib.html).

In principle, the batch-based design of CorALS also allows for more specialized implementations based on the MapReduce paradigm^41^. Thus, overall, CorALS provides a very flexible algorithmic framework for large-scale correlation analysis that can be easily extended and adjusted to the application at hand.

### Practical considerations

#### Full correlation matrix calculation.

On the basis of the results in Table 2 and Supplementary Table 3, where CorALS substantially outperforms all other methods, we recommend generally using CorALS for full correlation matrix calculation. As the number of features grows, however, the full correlation matrix will not fit into memory. For example, at n=32,000 features, the full matrix uses more than 8GB of memory; at n=64,000 features, it already requires more than 32GB. This can be calculated roughly by assuming 64-bit float values (default in Python) and the formula: memory $(n)=\frac{64n^{2}}{8\times 10^{9}}$. Thus, we recommend switching to top-*k* correlation analysis after n=32,000 features.

#### Top-*k* correlation search.

For top-*k* correlation search, we recommend using the basic CorALS implementation (referred to as matrix in Table 2) as long as the full correlation matrix fits into memory, independent of the number of samples. However, as the number of features increases, memory issues will make this approach impossible to use. When this is the case, switching to the index-based CorALS implementation is the best option. With increasing sample numbers, CorALS becomes slower, which may warrant other heuristics such as dimensionality reduction such as locality sensitive hashing or random projections (see ‘Discussion’).

Note that, by default, the top-*k* approximation approach does not guarantee symmetric results, that is, even if cor(x,y) is returned, cor(y,x) may be missing. This can be addressed by various post-processing steps, for example, by adding missing values. CorALS provides the option to enable this feature. In the experiments, this is not enabled as symmetric results are redundant for practical purposes.

#### Correlation structure visualization.

For practical purposes, there are two properties of the proposed correlation structure visualization to consider. First, by design, CorALS visualizes strongly positively correlated features close to each other while the distance to strongly negatively correlated features will be large (see corollary in ‘Correlation projections’). In some settings it may be desirable to simultaneously visualize negatively correlated features close to each other, which is currently not supported by CorALS. Second, the relationship between Euclidean distance and correlation established in is not linear, which may result in bias toward tightly clustering highly correlated features. See Supplementary Fig. 1 for an illustration of the relation between correlation and the corresponding Euclidean distance.

### Investigating the coordination of single-cell functions

For the analysis in ‘Correlated functional changes across immune cells’ and Fig. 3, we first divide cells into 20 individual non-overlapping cell types based on manual gating^1^. We then repeatedly sample 10,000 cells from each cell type across all patients using a dual bootstrapping scheme to ensure appropriate variations in cell types where less than 10,000 cells are present. The dual bootstrapping scheme first samples n cells from each cell type with replacement, where n is the number of available cells for that cell type. From this intermediate sample, we sample the final 10,000 cells for that cell type with replacement.

On the resulting sample of 200,000 cells across cell types, we calculate the top-0.01% Spearman correlations across all sampled cells based on their functional markers. We then count the number of top correlations between each pair of cell types. This allows to measure the relative correlation strengths between cell types.

By generating pairs of samples in each repetition, one from third-trimester cells and one from postpartum cells, we calculate the effect size (Cliff’*s δ*) of the top-*k* frequency differences between each pair of cell types. Supplementary Fig. 6 depicts a single instance of such a pair. We sample 1,000 times. Very large effect sizes defined by a corresponding effect size threshold (t=0.622) are visualized in Fig. 3. This threshold has been derived based on analogous interpretation intervals proposed for Cohen’s d (refs. 56,57).

As described above, this procedure requires repeated sampling and top-*k* correlation calculations across millions of individual cells, making CorALS an essential component of this pipeline, enabling this analysis on our available servers by substantially reducing runtime and particularly memory requirements.

### Datasets

The four real-world datasets we use for runtime and memory evaluation stem from biological applications in the context of pre-eclampsia, healthy pregnancy and cancer.All previously reported feature counts are subject to the following pre-processing procedure. We set negative values to 0, remove features that have only a single value and drop duplicate features (features are considered duplicates if all their sample values are the same). Dataset statistics are summarized in Table 1. For dataset availability, see Section ‘Data availability’.

The pre-eclampsia dataset^24,26^ contains aligned measurements from the immunome, transcriptome, microbiome, lipidome, proteome and metabolome, from 23 pregnant women with and without pre-eclampsia across the three trimesters of pregnancy. In brief, women of at least 18 years of age in their first trimester of a singleton pregnancy were recruited to the study after providing their informed consent and under institutional review board (IRB)-approved protocols. Whole blood, plasma and urine samples, and vaginal swabs were collected throughout pregnancy and processed to generate immunome, transcriptome, microbiome, lipidome, proteome and metabolome datasets. After aligning omics and dropping features with missing or only homogeneous values, 32 samples with 16,897 features where obtained.

The pregnancy dataset^6^ contains 68 samples from 17 pregnancies with four samples per woman in the first, second and third trimesters as well as postpartum, respectively. Each sample contains immunome, transcriptome, microbiome, proteome and metabolome measurements obtained simultaneously. In brief, women of at least 18 years of age in their first trimester of a singleton pregnancy were recruited to the study after providing their informed consent and under IRB-approved protocols. Whole blood, plasma and serum samples, and vaginal, stool, saliva and gum swabs were collected throughout pregnancy and processed to generate immunome, transcriptome, microbiome, proteome and metabolome datasets. After aligning omics and dropping features with missing or only homogeneous values, 32,211 features where obtained.

The cancer dataset contains samples from 443 patients with gastric adenocarcinoma^58^ and 185 patients with esophageal carcinoma^59^, for a total of 628 samples obtained via the LinkedOmics platform^25^. In brief, fresh frozen tumor samples and accompanying healthy tissue were collected from patients after providing their informed consent and under IRB-approved protocols. Samples were used to generate DNA methylation profiling at the CpG-site and gene levels (methylation CpG site level, HM450K; methylation gene level, HM450K), whole-exome sequencing (mutation gene level), messenger RNA sequencing (HiSeq, gene level), reverse-phase protein array (analyte level) and somatic copy number variation (gene level, log-ratio) datasets. After aligning omics and dropping features with missing or only homogeneous values, the dataset consisted of samples from 258 patients. For our runtime and memory experiments, we sample increasing numbers of features (25%,50% and 100%).

The single-cell datase^1^ contains 68 mass cytometry samples from 17 pregnancies with four samples per woman in the first, second and third trimesters as well as postpartum, respectively. In brief, women of at least 18 years of age in their first trimester of a singleton pregnancy were recruited to the study after providing their informed consent and under IRB-approved protocols. Whole blood samples were collected throughout pregnancy and processed to generate an immunome dataset. For the benchmark experiments, samples from the third trimester were used. We process the data by sampling 10,000/30,000 cells from each of the 20 cell types, resulting in a dataset with 200,000/600,000 cells and 10 functional markers per cell.

We also add one more dataset (sim) that corresponds to 400,000 features and 500 samples to test larger sample sizes. The data are generated randomly.

### Experimental settings for runtime and memory analysis

Experiments were repeated from 3 to 10 times depending on their runtime, the first sample was always dropped (to account for burn-ins, for example, for Julia’s JIT compiler), and respective medians are reported. No substantial runtime or memory fluctuations were observed. The experiments were run on a bare metal server with two AMD EPYC 7452 32-Core Processors and hyper-threading enabled amounting to 128 processing units. The machine provided 314GB of memory and ran on Ubuntu 20.04.1 LTS. We use Python 3.9.1 and R 4.0.3 with current packages installed from conda-forge and Bioconductor. The employed Julia version was 1.5.3. Multi-threading was disabled explicitly if not otherwise specified.

### Reporting summary

Further information on research design is available in the Nature Portfolio Reporting Summary linked to this article.

## Data availability

The pre-eclampsia dataset is available from a public repository^60^. The multiomics pregnancy dataset is available from a public repository^60^, and the original authors’ website^6^. Intermediate data to produce Fig. 2 are provided through a public repositor^60^. The cancer dataset is derived from a multiomics study available from LinkedOmics (http://linkedomics.org/data_download/TCGA-STAD/). In particular, we integrate the datasets methylation (CpG-site level, HM450K), methylation (gene level, HM450K), mutation (gene level), RNA sequencing (HiSeq, gene level), reverse-phase protein array (analyte level) and somatic copy number variation (gene level, log-ratio). The single-cell dataset used to derive the benchmark dataset single cell and to support the findings is available from FlowRepository (http://flowrepository.org/id/FR-FCM-ZY3Q). Pre-processed data for benchmarking as well as intermediate data to produce Fig. 3 are provided through a public repository^60^. We provide source data for all figures and tables, as well as download instructions and pre-processing scripts through a public repository^60,61^ and via https://nalab.stanford.edu/corals/.

## Code availability

The complete code for CorALS, code to reproduce all experiments and figures in this paper, and links and instructions to prepare the corresponding datasets are available in a public repository^61^, and are listed at https://nalab.stanford.edu/corals/.

## Supplementary Material

Supplementary material

## Figures and Tables

**Table 1 |:** Dataset statistics

Dataset	*n* (features)	*m* (samples)	*n/m* ratio
Pre-eclampsia	16,897	32	528
Pregnancy	32,211	68	474
Cancer (0.25)	64,813	258	251
Cancer (0.50)	129,626	258	502
Cancer (1.00)	259,252	258	1,005
Single cell	200,000	10	20,000
Single cell 2	600,000	10	80,000
Sim	200,000	500	400

Dimensions of biological datasets after pre-processing including feature-to-sample ratios. For this paper, CorALS is optimized to handle high-dimensional datasets, that is, for investigating correlations between n features based on m samples often with n≫m. Deviating from this terminology, note that for the single-cell dataset, correlations between individual cells (n) based on the expression of their functional markers (m) are investigated

**Table 2 |:** Top-*k* correlation network approximation

Dataset	Baselines			CorALS		
	R	Julia	Python	Matrix	Index	Parallel
Pre-eclampsia	33.6	1:41.9	1:11.6	6.3	14.3	2.4

Pregnancy	2:09.7	8:35.4	4:56.1	16:4	1:49.2	5.7

Cancer (0.25)	7:09:00.2	53:19.9	22:53.1	2:02.5	32:54.4	59.6

Cancer (0.50)	–	–	–	–	2:10:25.2	2:58.4

Cancer (1.00)	–	–	–	–	8:42:12.9	11:28.5

Single cell	–	–	–	–	16:10.1	1:46.9

Single cell 2	–	–	–	–	2:10:12.4	27:03.3

Sim	–	–	–	–	10:29:30.3	26:40.2

Pre-eclampsia	7.5 GB	6.4 GB	6.8 GB	6.8 GB	0.7 GB	3.4 GB

Pregnancy	27.3 GB	23.3 GB	23.7 GB	23.7 GB	1.3 GB	4.5 GB

Cancer (0.25)	158.2 GB	93.8 GB	94.3 GB	94.3 GB	4.1 GB	8.7 GB

Cancer (0.50)	**>0.7 Tb**	**>360 GB**	**>360 GB**	**>360 GB**	14.3 GB	21.1 GB

Cancer (1.00)	**>3.4 Tb**	**>1.4 Tb**	**>1.4 Tb**	**>1.4 Tb**	53.5 GB	65.2 GB

Single cell	**>1.9 Tb**	**>0.8 Tb**	**>0.8 Tb**	**>0.8 Tb**	33.2 GB	38.7 GB

Single cell 2	**>23.3 Tb**	**>7.9 Tb**	**>7.9 Tb**	**>7.9 Tb**	253.1 GB	281.3 GB

Sim	**>1.9 Tb**	**>0.8 Tb**	**>0.8 Tb**	**>0.8 Tb**	31.1 GB	36.5 GB

Runtime and memory comparison. The runtime (top half of table; hours:minutes:seconds) and memory (bottom half of table; GB or Tb) comparison for top-*k* correlation network approximation (k=0.1% of features). Dashes represent the lack of runtime measurements for examples exceeding our server resources. Bolded entries mark estimated memory consumption for examples exceeding our server resources.
