# Supplementary material for: Large-scale correlation network construction for unraveling the coordination of complex biological systems
Source: Nat Comput Sci. Author manuscript; Available in PMC 2024 Apr 13. (PMC10727505; doi:10.1038/s43588-023-00429-y)
Supplement: Supplementary material [file NIHMS1902999-supplement-Supplementary_material.pdf]

nature computational science

Resource

https://doi.org/10.1038/s43588-023-00429-y

Large-scale correlation network construction for unraveling the coordination of complex biological systems

In the format provided by the authors and unedited

Supplementary Information

I Supplementary Material 3

Supplementary Section 1 Runtime and memory profiles on synthetic data 3

Supplementary Section 1.1 Full correlation matrix calculation 3

Supplementary Section 1.2 Top-k correlation search 4

Supplementary Section 2 Runtime profiles with multi-core processing 5

Supplementary Section 2.1 Full correction matrix calculation 5

Supplementary Section 2.2 Top-k correlation network approximation 5

Supplementary Section 2.3 Top-k differential correlation network approximation . 6

Supplementary Section 3 Comparison with existing software libraries 6

Supplementary Section 3.1 Computation of full correlation matrices 6

Supplementary Section 3.2 Computation of top-k correlation networks 7

Supplementary Section 3.3 Differential analysis of correlation networks 8

Supplementary Section 4 Time and space complexity analysis 9

Supplementary Section 4.1 Computation of correlation matrices 9

Supplementary Section 4.2 Estimation of large-scale correlation networks 10

Supplementary Section 4.3 Differential analysis of correlation networks 11

Supplementary Section 5 Approximation properties 12

Supplementary Section 5.1 Optimal approximation factor 12

Supplementary Section 5.2 Experimental approximation properties 20

Supplementary Section 6 Details on the feature representations employed by *CorALS* 21

Supplementary Section 6.1 Correlation projections 21
Supplementary Section 6.2 Differential projections 22

Supplementary Section 7 Performance of correlation embeddings 25

Supplementary Section 8 Addendum to ”Large-scale multiomics correlation analysis across pregnancy” 26

Supplementary Section 9 Addendum to ”Correlated functional changes across immune cells” 28

Supplementary Section 10 A brief history on the correlation coefficient 30

II Supplementary Algorithms 33

III Supplementary Figures and Tables 34

Part I
Supplementary Material

Supplementary Section 1 Runtime and memory profiles on synthetic data

In order to investigate runtime and memory profiles of *CorALS* in more detail, we conduct a set of experiments on synthetic data. The basic synthetic dataset is a random matrix with 20,000 features (columns) and 50 samples (rows). In the following experiments, we gradually add more features and samples, respectively, and analyze the results.

Supplementary Section 1.1 Full correlation matrix calculation

Figure 2 shows the runtime comparison of full correlation matrix calculation on synthetic data. The baseline data contains 20,000 features and 50 samples indicated by vertical gray lines. We then gradually modify the number of features and samples along the x-axis, respectively. The results confirm the trends from real-world data in the main text and exemplify how *CorALS* outperforms existing methods (default) in each programming language. In particular, as the number of features increases, the performance gain grows exponentially as illustrated by the constant distance of default and *CorALS* implementations (fast) on the log-log scale (see Figure 2a). Similarly, while common programming frameworks are more efficient in cases with low feature to sample ratios, when reducing the number of samples, the performance gain of *CorALS* becomes increasingly prominent (see Figure 2b). This illustrates that common programming frameworks are not optimized for scenarios with high feature to sample ratios, and that *CorALS* effectively tackles this issue to enable large-scale correlation analysis in high-dimensional biological datasets, e.g., in multiomics or single-cell settings.

Supplementary Section 1.2 Top-k correlation search

The runtime results for the top-k correlation matrix approximation on synthetic data are shown in Figure 3. The baselines for R, Julia, and Python are implemented by calculating the full correlation matrix, sorting the corresponding absolute correlation values (using respective default sorting implementations), and selecting the top k entries. The generally longer runtimes of extracting top k correlations compared to calculating the full correlation matrix in Supplementary Section 1.1 is due to the time intensive sorting step required to identify the most prominent correlations. Figures 3a and 3b show that for data with high feature to sample ratios, taking advantage of the advanced indexing structures (*CorALS*) yields substantial performance gains over a sorting-based implementation (default). With an increasing number of features, the constant difference in runtime in the log-log plot implicates exponentially growing performance gains. At the same time decreasing samples counts illustrate how *CorALS* can substantially outperform naive implementations for high feature to sample ratios. It is important to note, that with growing sample counts the data space becomes sparser (curse-of-dimensionality) which can reduce the performance of the tree-based indexing method used by *CorALS* to degrade to linear runtimes, thus, reducing its runtime advantage. However, the inherent parallelization capabilities of the tree-based approach can mitigate this effect. Also, future optimizations, e.g., based on approximate nearest neighbor search like locality sensitive hashing (*1*), may further improve runtime performance in these scenarios. Furthermore, a particular strength of the *CorALS* implementation is its memory footprint as illustrated by Figure 3c which can enable top-k correlation network construction even on standard laptop computers.

Note that the Python implementation takes advantage of the dualtree approach (*2*) for speeding up the search. This specialization was only available for the Python implementation of ball trees (`sklearn`) but not for the Julia implementation (`NearestNeighbors.jl`). Thus, Julia has the potential to gain a performance boost for top correlation extraction. Finally, while

several k-nearest-neighbor libraries exist, no R library providing ball tree data structure was available to the authors at the time of writing this manuscript, which is why no corresponding implementation was included.

Supplementary Section 2 Runtime profiles with multi-core processing

The methods provided by *CorALS*, e.g., full correlation matrix calculation, top-k correlation network estimation, are highly parallelizable. This is illustrated in Figure 4 on the Cancer (0.25) dataset. Similar characteristics apply for differential correlation discovery. This illustrates the versatility of *CorALS* and further outlines the advantage over common implementations in state-of-the-art programming frameworks for data with high feature to sample ratios.

Supplementary Section 2.1 Full correction matrix calculation

Since the full correlation matrix calculation is based on matrix multiplication, it is inherently parallelized by the underlying matrix routines employed by the different programming frameworks. In previous experiments, we have specifically disabled this inherent parallelization. Figure 4a shows the effect of using multiple cores on the full correlation matrix calculation. Substantial speedups can be achieved. Note that, for this, it can be important to multiply two physical copies of the original sample-feature matrix as more cores are utilized to gain the most speedup (compare the runtime of CorALS *copy* vs. CorALS *no copy* in Figure 4a).

Supplementary Section 2.2 Top-k correlation network approximation

Additionally, the top k correlation network approximation is inherently parallelizable by concurrently querying the underlying index structure. This also results in substantial speedups as the number of used processing units increases as depicted in Figure 4b while the memory overhead is relatively small.

Supplementary Section 2.3 Top-k differential correlation network approximation

The parallelization properties of differential correlation discovery is equivalent to top-k correlation network approximation.

Supplementary Section 3 Comparison with existing software libraries

Supplementary Section 3.1 Computation of full correlation matrices

As mentioned in the main manuscript, R in particular has numerous different implementations to calculate full correlation matrices, likely motivated by the limited performance of the native `cor` function. This includes packages like WGCNA (*3*), Rfast (*4*), coop (*5*), and HiClimR (*6*) with different advantages and disadvantages. For example, WGCNA can handle small amounts of missing values, and HiClimR tries to save memory by only calculating the upper half of the correlation matrix. Supplementary Data 1 shows a performance comparison on a subset of real-world datasets, illustrating that *CorALS* outperforms the other implementation, in most cases with substantial performance gains.

Similarly, the Python ecosystem provides several packages that allow for efficient correlation matrix calculation. This includes for example the DeepGraph package (*7*), Dask (*8*), or Spark (*9*) However in initial tests, did not come close to the runtime performance provided by *CorALS*. For reference, we used a synthetic dataset with 32 211 features and 68 samples which is equivalent to the pregnancy dataset (`PREG`). In this setting, and utilizing 64 cores, DeepGraph takes 1 minute and Dask requires 11 seconds. For Spark, the `pyspark.ml.stat.Correlation` takes about 7 seconds to finish. All are slower than the *CorALS* implementation which only needs roughly 1 second. DeepGraph may be useful when memory resources are limited since results are directly written to disc. Similarly, Dask supports spilling results to disk which do not fit into memory. By design, Spark keeps all correlations in memory, but allows to keep

them distributed if set up accordingly. However, in contrast to *CorALS*, all above mentioned libraries require a substantial programming overhead as well as in-depth knowledge about parallel processing.

In addition to these two frameworks, Python libraries exist that allow for utilizing GPUs for correlation computation. This includes for example CuPy (*10*), or RAPIDS (*11*). While both have the potential to rapidly speed up the calculation of the overall correlation matrix (e.g., for the previous scenario CuPy has similar runtimes as *CorALS* of around 1 second), their memory footprint is inherently limited by the available GPU memory which in most cases is substantially smaller than main memory. In theory, combinations of Dask, CuPy, and RAPIDs are possible to enable distributing workload across GPUs or utilize a batched approach, however this does not represent a drop-in replacement and simple interface provided by *CorALS* and requires knowledge of the underlying parallelization framework.

Beside R and Python packages there is a variety of other implementations of efficient correlation matrix calculation (e.g., (*12*)) but they are often not easy to use, and similar to the previously mentioned approaches do not directly support the selection of top-k correlations as provided by *CorALS*. For example, some rely on specialized parallel processing and high performance computing frameworks (e.g., GPUs, MapReduce, etc.) (*13–16*) which *CorALS* supports through `joblib` backends. Also, the corresponding implementations are not easily accessible which is why we do not compare against these methods. Other methods focus on specialized correlation measures (*17, 18*), such as partial correlation or extract top-k correlations of pairs items in databases.

Supplementary Section 3.2 Computation of top-k correlation networks

To the best of the author's knowledge, no implementations for top-k correlation discovery exist. In particular, none of the methods for full correlation matrix calculation, mentioned in the previ-

ous section, support the efficient discovery of top-k correlations for a large-scale datasets. This includes the mentioned R implementations as well as Python based approaches like DeepGraph or Dask (`https://github.com/dask/dask/issues/2859`).

Supplementary Section 3.3 Differential analysis of correlation networks

There are a variety of methods for differential network analysis (*19–25*). While methods like Discordant (*24*) and DCARS (*25*) can be used to calculate differential correlations, do not allow for top-k functionality and thus will quickly run into memory issues. The closest to *CorALS*'s capability to derive top-k differential correlations are DGCA and DiffCorr (*19, 23*). However, when testing runtimes, DGCA was substantially slower: for a synthetic dataset of 10000 features and 17 samples across two conditions, DGCA ran 2.2 minutes while *CorALS* required 7.5 seconds (no parallelization, i.e., using a single core). DGCA, provide sampling functionality for more robust difference estimation which causes longer runtimes and has been disabled for comparability in this example. Similarly, DiffCorr, even with disabled adjustment, also exhibits substantially longer runtimes (1.5 minutes) for the same dataset. Similar sampling techniques can be implemented in *CorALS* as exemplified in the *Main Article*, "CorALS reveals changes in correlations across signaling pathways in immune cell types". Alternatively, *CorALS* can be used as an efficient candidate selection step, and the results can then potentially be tested for their robustness using one of the previously mentioned more robust approaches.

Other methods are based on different network comparison concepts. This includes for example chNet (*21*), which incorporates partial correlation and hierarchical properties. From a performance perspective, it runs more than 5 minutes on a network with only 1000 features which is substantially slower that the previously mentioned methods (e.g., chNet runs more than 5 minutes on 1000 features while *CorALS* only run 7.5 seconds on 10000 features). Additionally, while DINGO (*22*) looks comparable to *CorALS* and even incorporates pathway

analysis into the differential analysis process, unfortunately, no well documented implementation was found to evaluate against. Finally, BioNetStat (*20*) compares overall correlation networks based on centrality and allows to compare across more than two networks, but exhibits runtimes well beyond 3 minutes for a dataset with 10000 features which is substantially slower than *CorALS* at 7.5 seconds (no parallelization, i.e., using a single core). Overall, while there are many methods and flavors available for differential network analysis, they all perform substantially worse with regard to runtime as outlined above and/or are equally expected to suffer from memory restrictions. Thus, as some of the discussed packages are frameworks for more advanced analysis capabilities, *CorALS* holds substantial potential to further speed up the corresponding algorithms.

Supplementary Section 4 Time and space complexity analysis

Supplementary Section 4.1 Computation of correlation matrices

Naive. The naive implementation to calculate a correlation matrix consists of two nested loops along the n_1 columns of matrix D_1 and the n_2 columns for matrix D_2. In the context of *CorALS*, n_i represents the number of features in the data matrix D_i. For the sake of the following derivation, we assume $D_1 = D_2$ and thus $n := n_1 = n_2$. In each iteration, the correlation is then calculated using a dot product across the shared number of rows m. This results in a theoretical runtime of $\mathcal{O}(n^2 \cdot m)$. The space complexity of this computation is based on the size of the matrix holding all computed correlations $\mathcal{O}(n^2)$. Note that this space complexity causes full correlation matrix calculations to quickly exceed memory resources as exemplified in our experiments (see Supplementary Data 1).

CorALS. For full correlation matrix calculation, *CorALS* has the same time and space complexity as the naive approach. Like other methods (e.g., `coop` (*5*)), *CorALS* relies on efficiently

implemented matrix multiplication routines to speed up the calculation of the correlation matrix. The Python implementation of *CorALS* is based on `numpy` (currently version `1.20.3` which uses BLAS and LAPACK routines (*26, 27*) (optionally multi-threaded). In practical settings, this implementation outperforms other implementations which we evaluate empirically (see Supplementary Data 1).

Other methods. Other methods either use efficient custom C implementations which are often based on (multi-threaded) nested for-loops (like, e.g., `Rfast` (*4*)) or use efficient BLAS and LAPACK routines (like `coop` (*5*)) similar to *CorALS*. As such their runtime heavily depends on specific implementation details and, thus, has to be evaluated and compared against *empirically* (see Supplementary Section 3). Analogously to *CorALS*, their space complexity is defined by the resulting correlation matrix and thus is $\mathcal{O}(n^2)$.

Supplementary Section 4.2 Estimation of large-scale correlation networks

Naive. The naive implementation of calculating top-k correlations first (i) calculates the full correlation matrix, then (ii) sorts correlations, and finally (iii) selects and returns the top-k correlation values. Calculating the full correlation matrix has time complexity of $\mathcal{O}(n^2 \cdot m)$ (see above) and space complexity of $\mathcal{O}(n^2)$. Sorting all n^2 correlations, e.g., with Quicksort, then amounts to a runtime complexity of $\mathcal{O}(n^2 \cdot log(n^2))$ (*28*). The selection step (e.g., based on Introselect (*29*)) after sorting has runtime complexity of $\mathcal{O}(n^2)$. The main driver of time complexity is the sorting step after calculating the full correlation matrix with $\mathcal{O}(n^2 \cdot log(n^2))$. The main cost in memory consumption is caused by having to calculate the full correlation matrix with space complexity of $\mathcal{O}(n^2)$.

CorALS. *CorALS* addresses the previously mentioned main drivers of time and space complexity by avoiding calculating the full correlation matrix (step (i)) while at the same time substantially reducing the number sorted values (step (ii)). For this, *CorALS* first derives a

space partitioning index structure (*30*). This has a time complexity of $\mathcal{O}(n \cdot log(n))$ and a space complexity of $\mathcal{O}(n)$ (*31*). Then, searching for the top-1 correlation for *a single feature* has a time complexity of $\mathcal{O}(log(n))$ on average but can degrade to linear runtime with increasing numbers of samples m with optimal performance if $2^m << n$ (*31*). In the following, we assume the average case and $2^m << n$ in order to illustrate the practical performance advantage of *CorALS*. Furthermore, searching for top-$(a\frac{k}{n})$ correlations for *a single feature* has a time complexity of $\mathcal{O}(log(a\frac{k}{n}) \cdot log(n))$ on average, where a is the approximation factor (see Supplementary Section 5) and k is the number of overall correlations to search for. Searching for the top-$(a\frac{k}{n})$ values across all features then results in an overall average time complexity of $\mathcal{O}(n \cdot log(a\frac{k}{n}) \cdot log(n))$. This search step then results in $n \cdot (a\frac{k}{n}) = a \cdot k$ correlation values. These correlations are then sorted and the top-k correlations are returned. Then, the sorting step has a time complexity of $\mathcal{O}(ak \cdot log(ak))$. Depending on how k is selected, the theoretical time complexity analysis can vary, e.g., if k is a constant the time complexity is $\mathcal{O}(1)$, if k is selected depending on the number of features n, time complexity is $\mathcal{O}(n \cdot log(n))$, and if k is selected based on the overall number of possible correlations n^2, time complexity is $\mathcal{O}(n^2 \cdot log(n))$. In any case, the time complexity is substantially lower than $\mathcal{O}(n^2 \cdot log(n^2))$ of the naive approach. Consequently, practical run times can differ based on the dataset and parameters selected by the user. Similarly, the space complexity of *CorALS* is dependent on the approximation factor and selected k: $\mathcal{O}(a \cdot k)$.

Supplementary Section 4.3 Differential analysis of correlation networks

Time and space complexity analysis of top-k differential correlation analysis is generally analogous to top-k correlation analysis. For the naive implentation, two full correlation matrices are calculated (instead of one) and subtracted. The subsequent steps to extract differntial correlations is then equivalent to top-k correlation calculation. For *CorALS* differential vectors

are constructed that replace the correlation projections (see *Main Article*, ”Differential projections”). After that, indexing, search, and sorting is equivalent to *CorALS*’s top-k correlation calculation approach.

Supplementary Section 5 Approximation properties

Supplementary Section 5.1 Optimal approximation factor

CorALS searches for the top-k correlations by extracting $k' = a\frac{k}{n}$ top correlation candidates per feature and then merging the results across all n features. However, because top-k correlations may not be equally distributed across all features (i.e., $\frac{k}{n}$ top-k correlation per feature), *CorALS* introduces the approximation factor a that allows to specify a safety margin for cases were more than $\frac{k}{n}$ top-k correlations are associated with a single feature. In the following, we examine how the approximation factor a influences the accuracy of *CorALS* for returning top-k correlations. Note that we always assume $k \geq n$.

First, if top-k correlations are equally distributed across all features, $a = 1$ is sufficient. If this is not the case, we show in Theorem 5.3 the maximum number of top-k correlations missed as a function of a given *correlation-per-feature* threshold (where a threshold of t means we extract the top t correlations per feature by *CorALS*). Based on this, one can choose a threshold that will return the top-k correlations with a desired minimum sensitivity, where the exact sensitivity of *CorALS* will depend on the correlation matrix itself.

Notation. For the following theorems, we first define a set of notations: Let n be the number of features, and K be the set of top-k correlations, with $k = |K| \leq n^2$. Furthermore, let $k_i = |K_i|$ denote the number of *local* top-k correlations $c_{i,\cdot}$ contributed by feature f_i, with $K_i = \{c_{ij} \mid c_{ij} \in K\}$. Let F be the set of features with at least one correlation in K, i.e., $F = \{f_i \mid |K_i| \geq 1\}$ Also, without loss of generality, let $\forall i < j : k_i \geq k_j$, i.e., let f_1 contribute the largest number of correlation, f_2 the second largest (or equal), and so on. Additionally, let $r_i(c_{ij})$ denote the

rank of c_{ij} across all correlations for feature f_i, i.e., $r_i(c_{ij}) < r_i(c_{ik}) \Rightarrow |c_{ij}| > |c_{ik}|$, and let $K_{i|r_i \leq t} = \{c_{ij} \mid c_{ij} \in K \wedge r_i(c_{ij}) \leq t\}$ denote the *t-ranked* local top-k correlations. For this, the threshold t denotes the number of top t correlations extracted per feature by *CorALS*, and r_i represents the rank of all c_{ij} in $K_{i|r_i \leq t}$, i.e., which have a rank $r_i(c_{ij})$ smaller or equal to t. Also, we define $k_{i|r_i \leq t} = |K_{i|r_i \leq t}|$ and thus $k_{i|r_i \leq t} = min\{t, k_i\}$. Note that with $K' = \bigcup_{i=1}^{n} K_{i|r_i \leq a\frac{k}{n}}$, the set $K' \cup \{c_{ji} \mid c_{ij} \in K'\}$ is exactly the set of *correct* top-k correlations found by *CorALS*, when taking advantage of the symmetry of correlations. Furthermore, we define the set of *t-constrained* local top-k values as $K_{i|j \leq t} = \{c_{ij} \mid c_{ij} \in K \wedge j \leq t\}$ and $k_{i|j \leq t} = |K_{i|j \leq t}|$, as those top-$k$ correlations associated with at least one of first t features ($j \leq t$). Consequently, note that given any feature f_i, the number of t-constrained features can not exceed t: $\forall i : k_{i|j \leq t} \leq t$. Finally, let $k_{\text{found}}(t)$ and $k_{\text{missed}}(t)$ be the number of top-k correlations found and missed by *CorALS* respectively, such that $k = k_{\text{found}}(t) + k_{\text{missed}}(t)$.

Lemma 5.1. ***Minimum Top-k Correlations.*** *For a threshold t,* CorALS *finds at least* $\sum_{i=1}^{n} k_{i|r_i \leq t} \geq \sum_{i=1}^{n} k_{i|j \leq t}$ *top-k correlations. That is* $k_{found}(t) \geq \sum_{i=1}^{n} k_{i|r_i \leq t} \geq \sum_{i=1}^{n} k_{i|j \leq t}$.

Proof. Given a threshold t, by definition *CorALS* extracts $k_{i|r_i \leq t}$ top-k correlations for each feature f_i resulting in the overall number of found correlations of $\sum_{i=1}^{n} k_{i|r_i \leq t}$. When exploiting the symmetry of the correlation matrix, the number of found correlations may be larger. Thus, $k_{\text{found}}(t) \geq \sum_{i=1}^{n} k_{i|r_i \leq t}$. Furthermore, since t-constrained top-k correlations are restricted to correlations involving at least one feature f_j up the threshold $j \leq t$, while t-ranked features span any features, it holds that $\forall i : k_{i|i \leq t} \leq k_{i|r_i \leq t} = min\{t, k_i\}$. Thus, $k_{\text{found}}(t) \geq \sum_{i=1}^{n} k_{i|r_i \leq t} \geq \sum_{i=1}^{n} k_{i|j \leq t}$.

Lemma 5.2. ***Symmetry.*** *Let t be a threshold with* $1 \leq t \leq n, t \in \mathbb{N}$. *Then the number of top-k correlations contributed by the first t features, i.e.,* $\sum_{i=1}^{t} k_i$*, is i) equal to the number of* t-constrained *local top-k correlations summed across all features, i.e.,* $\sum_{i=1}^{t} k_i = \sum_{i=1}^{n} k_{i|j \leq t}$,

and ii) smaller than or equal to the number of t-ranked *local top-k correlations summed across all features, i.e.,* $\sum_{i=1}^{t} k_i \leq \sum_{i=1}^{n} k_{i|r_i \leq t} = \sum_{i=1}^{n} min\{t, k_i\}$.

Proof. For i) let us consider the set of correlations $K_{i\leq t}$ contributed by the first t features. It holds that:

$$K_{i\leq t} = \bigcup_{i=1}^{t} K_i = \bigcup_{i=1}^{t} \{c_{i,\cdot} \mid c_{i,\cdot} \in K_i\} = \bigcup_{i=1}^{t} \{c_{i,\cdot} \mid c_{i,\cdot} \in K \wedge i \leq t\} = \{c_{i,\cdot} \mid c_{i,\cdot} \in K \wedge i \leq t\}$$

Note that $|K_{i\leq t}| = |\bigcup_{i=1}^{t} K_i| = \sum_{i=1}^{t} k_i$. Now, let $\overline{K}_{i\leq t} = \{c_{j,\cdot} \mid c_{\cdot,j} \in K_{i\leq t}\}$ be the set of "transposed" correlations. By definition these two sets have the same cardinality: $|K_{i\leq t}| = |\overline{K}_{i\leq t}|$, and when exploiting the symmetry of correlation matrices, i.e., $c_{ij} = c_{ji}$, the correlations in $\overline{K}_{i\leq t}$ are also part of the top-k correlations, i.e., $\overline{K}_{i\leq t} \subseteq K$. Based on this:

$$\begin{aligned} \sum_{i=1}^{t} k_i &= |K_{i\leq t}| = |\overline{K}_{i\leq t}| = |\{c_{j,\cdot} \mid c_{\cdot,j} \in K_{i\leq t}\}| \\ &= |\bigcup_{j=1}^{n} \{c_{ji} \mid c_{ij} \in K_{i\leq t}\}| = |\bigcup_{j=1}^{n} \{c_{ji} \mid c_{ij} \in K \wedge i \leq t\}| \\ &= |\bigcup_{i=1}^{n} \{c_{ij} \mid c_{ij} \in K \wedge i \leq t\}| = \sum_{j=1}^{n} k_{j|i\leq t} = \sum_{i=1}^{n} k_{i|j\leq t} \end{aligned}$$

Then ii) directly follows from i) as $\forall i : k_{i|i\leq t} \leq k_{i|r_i\leq t}$ (also see proof for Theorem 5.1).

Theorem 5.3. *Given a threshold t,* CorALS *finds* $k_{i|r_i\leq t} = min\{t, k_i\}$ *top-k correlations for each feature f_i. This may result in missed top-k correlations:* $k = k_{found}(t) + k_{missed}(t)$. *Based on the threshold used, the number of found correlations is always at least:*

$$k_{found}(t) \geq \begin{cases} t\sqrt{k} & for\ 1 \leq t \leq \frac{3}{4}\sqrt{k} \\ 2t(\sqrt{k+t^2} - t) & for\ \frac{3}{4}\sqrt{k} \leq t \end{cases}$$

Proof. Let $T(t)$ be the number of features that contribute more or equal to t correlations to K, i.e., $\forall i \leq T(t) : k_i \geq t$ and $T(t) = |\{i \mid k_i \geq t\}|$. Let us note that for two thresholds t_1 and t_2 with t_1 smaller than t_2, i.e., $t_1 < t_2$, we must have that i) the number of features contributing

at least t_1 top-k correlations is greater or equal to the number of features contributing at least t_2 top-k correlations, i.e., $T(t_1) \geq T(t_2)$, ii) the number of found top-k correlations is smaller for t_1 than for t_2, i.e., $k_{\text{found}}(t_1) \leq k_{\text{found}}(t_2)$, and iii) the number of missed correlations is larger for t_1 than for t_2, i.e., $k_{\text{missed}}(t_1) \geq k_{\text{missed}}(t_2)$. Finally, given the definition of $T(t)$, we must also have that $k_{\text{found}}(t) \geq tT(t)$ and that *CorALS* finds all the top correlations of features f_i with $i > T(t)$.

Next, we will derive two lower bounds on the number of found top-k correlations. For the first bound, we will take advantage of the fact that *CorALS* utilizes the symmetry of the correlation matrix to infer top correlations which might otherwise have been missed due to the threshold used. Specifically, a top correlation c_{ij} might be missed by *CorALS* when looking at the top correlations of feature f_i, but if it is found when looking at the top correlations of feature f_j as c_{ji}, then *CorALS* will in essence also return c_{ij}. Given that *CorALS* will always return all top-k correlations located in features f_i with $i > T(t)$, all correlations which are ultimately missed by *CorALS* must be between features f_i and f_j with $1 \leq i, j \leq T(t)$. Since there are at most $T(t)^2$ of these correlations, we must have that $k_{\text{missed}}(t) \leq T(t)^2$ and thus we have a first lower bound on the found top-k correlations: $k_{\text{found}}(t) \geq k - T(t)^2$.

We now derive a second lower bound on the number of found top-k correlations, i.e., we show that $k_{\text{found}}(t) \geq \frac{1}{2}k + tT(t) - \frac{1}{2}T(t)^2$. To show this, we will be looking at the correlations found by *CorALS before* leveraging the symmetry of the correlation matrix to fill in possibly-missed correlations. In other words, let $k'_{\text{found}}(t)$ and $k'_{\text{missed}}(t)$ be the number of top-k correlations found by *CorALS* before filling in correlations via symmetry. Naturally, we have $k'_{\text{found}}(t) \leq k_{\text{found}}(t)$ and $k'_{\text{missed}}(t) \geq k_{\text{missed}}(t)$ since the filling-in process can only increase the number of found top-k correlations. Now, let us first note that from Theorem 5.1 and Theorem 5.2, $k'_{\text{found}}(T(t)) \underbrace{\geq}_{Theorem\ 5.1} \sum_{i=1}^{n} k_{r_i \leq T(t)} \underbrace{\geq}_{Theorem\ 5.2} \sum_{i=1}^{T(t)} k_i = tT(t) + k'_{\text{missed}}(t)$. The last equality is valid since by definition of $T(t)$ the first $T(t)$ features contribute at least t top-k

correlations. We will use this inequality for the following two cases:

Case 1: $t \leq T(t)$.

First we note that the top-k correlations found using a threshold of t are a subset of the top-k correlations found using the larger threshold of $T(t)$. Additionally, by definition of $T(t)$, the correlations not found using threshold t but found by using threshold $T(t)$ must only involve the first $T(t)$ features (all other features contribute less than t top-k correlations and thus every corresponding top-k correlation will be found using a threshold of t). Finally, for these first $T(t)$ features, a threshold of $T(t)$ can only find up to $T(t) - t$ correlations that are not found by threshold t. Thus, we get that $k'_{\text{found}}(T(t)) \leq k'_{\text{found}}(t) + (T(t) - t)T(t)$. Additionally, due to Theorem 5.2 (with threshold $T(t)$), it holds that $k'_{\text{found}}(T(t)) \geq tT(t) + k'_{\text{missed}}(t)$. Together we get

$$
\begin{aligned}
k'_{\text{found}}(t) + T(t)(T(t) - t) &\geq k'_{\text{found}}(T(t)) \\
&\geq tT(t) + k'_{\text{missed}}(t) \\
&= tT(t) + k - k'_{\text{found}}(t) \\
\Rightarrow k'_{\text{found}}(t) &\geq \frac{1}{2}k + tT(t) - \frac{1}{2}T(t)^2
\end{aligned}
$$

as wanted.

Case 2: $T(t) \leq t$.

Reversed to the previous case, here, we note that the top-k correlations found using a threshold of $T(t)$ are a subset of the top-k correlations found using the larger threshold of t. Additionally, by definition of $T(t)$, the first $T(t)$ features contribute at least t top-k correlations. Thus, we find at least $t - T(t)$ more top-k correlation for each of these first $T(t)$ features, when using a threshold of t rather than $T(t)$. Thus, we have that $k'_{\text{found}}(t) \geq k'_{\text{found}}(T(t)) + T(t)(t - T(t))$.

This means that

$$\begin{aligned} k'_{\text{found}}(t) &\geq T(t)(t - T(t)) + k'_{\text{found}}(T(t)) \\ &\geq T(t)(t - T(t)) + T(t)t + k'_{\text{missed}}(t) \\ &= 2T(t)t - T(t)^2 + k - k'_{\text{found}}(t) \\ \Rightarrow k'_{\text{found}}(t) &\geq \frac{1}{2}k + tT(t) - \frac{1}{2}T(t)^2 \end{aligned}$$

as wanted.

Finally, since $k_{\text{found}}(t) \geq k'_{\text{found}}(t)$, we must have that $k_{\text{found}}(t) \geq \frac{1}{2}k + tT(t) - \frac{1}{2}T(t)^2$ as wanted. This gives us two lower bounds for $k_{\text{found}}(t)$, so that

$$k_{\text{found}}(t) \geq \max(k - T(t)^2, \frac{1}{2}k + tT(t) - \frac{1}{2}T(t)^2)$$

and we can determine which of these bounds holds by analyzing how these two functions behave in terms of the number of features contributing more than t top-k correlations, i.e., $T(t)$. We can see that $k - T(t)^2 = \frac{1}{2}k + tT(t) - \frac{1}{2}T(t)^2$ when $T(t) = \pm\sqrt{t^2 + k} - t$. Since $T(t) \geq 0$, this means these functions are equal when $T(t) = \sqrt{t^2 + k} - t$. When $T(t) \leq \sqrt{t^2 + k} - t$, the first function is greater and $k_{\text{found}}(t) \geq k - T(t)^2 \geq k - (\sqrt{t^2 + k} - t)^2 = 2t(\sqrt{k + t^2} - t)$. When $T(t) \geq \sqrt{t^2 + k} - t$, the second function is greater and $k_{\text{found}}(t) \geq \frac{1}{2}k + tT(t) - \frac{1}{2}T(t)^2 \geq \frac{1}{2}k + t(\sqrt{t^2 + k} - t) - \frac{1}{2}(\sqrt{t^2 + k} - t)^2 = 2t(\sqrt{k + t^2} - t)$. Thus, overall, we have $k_{\text{found}}(t) \geq 2t(\sqrt{k + t^2} - t)$ whether $t \leq T(t)$ or $T(t) \leq t$.

Finally, we will determine when our bound holds for specific values of t. For this, we first note that when $T(t) \geq \sqrt{k}$, we will have that $k_{\text{found}}(t) \geq tT(t) \geq t\sqrt{k}$, since there are $T(t)$ features with at least t top-k correlations all of which are found by *CorALS*. In particular, for $t = 1$, we also have that $T(1) \geq \sqrt{k}$. Since $T(1)$ is the number of features with at least 1 correlation in K, we have that $T(1) = |F|$. However, since a set of F features has less than $|F|^2$ correlations total, we must have $|F|^2 \geq k$. Thus, $T(1) = |F| \geq \sqrt{k}$.

Now, we have shown that for $T(t) \geq \sqrt{k}$, $k_{\text{found}}(t) \geq t\sqrt{k}$ and for all t, $k_{\text{found}}(t) \geq 2t(\sqrt{k+t^2} - t)$. However, for a given correlation matrix, we don't know at which t we will have that $T(t) \geq \sqrt{k}$ and at which t we have that $T(t) \leq \sqrt{k}$. However, we will show that regardless of which of these two holds for a given t, $k_{\text{found}}(t) \geq \min(2t(\sqrt{k+t^2} - t), t\sqrt{k})$ always holds.

First, let's note that $\min(2t(\sqrt{k+t^2} - t), t\sqrt{k}) = t\sqrt{k}$ for $1 \leq t \leq \frac{3}{4}\sqrt{k}$ and $\min(2t(\sqrt{k+t^2} - t), t\sqrt{k}) = 2t(\sqrt{k+t^2} - t)$ for $t \geq \frac{3}{4}\sqrt{k}$. This can be seen by defining $t = r\sqrt{k}$ and solving

$$\begin{aligned}
t\sqrt{k} \leq 2t(\sqrt{k+t^2} - t) &\Leftrightarrow r\sqrt{k}\sqrt{k} \leq 2r\sqrt{k}(\sqrt{k + (r\sqrt{k})^2} - r\sqrt{k}) \\
&\Leftrightarrow rk \leq 2r\sqrt{k}(\sqrt{k + r^2 k} - r\sqrt{k}) \\
&\Leftrightarrow rk \leq 2rk\sqrt{1+r^2} - 2r^2 k \\
&\Leftrightarrow 1 \leq 2\sqrt{1+r^2} - 2r \\
&\Leftrightarrow 2r + 1 \leq 2\sqrt{1+r^2} \\
&\Leftrightarrow 4r^2 + 4r + 1 \leq 4 + 4r^2 \\
&\Leftrightarrow 4r + 1 \leq 4 \\
&\Leftrightarrow r \leq \frac{3}{4}
\end{aligned}$$

Now, let t' be the largest threshold such that $T(t') \geq \sqrt{k}$. Since $t'T(t') \leq k \leq \sqrt{k}T(t')$, we must have $t' \leq \sqrt{k}$. Thus, by definition of t', it holds that for a threshold $1 \leq t \leq t'$, $k_{\text{found}}(t) \geq t\sqrt{k}$, and for $t \geq t'$, $k_{\text{found}}(t) \geq 2t(\sqrt{k+t^2} - t)$. Next we compare the actual found values to what $\min(2t(\sqrt{k+t^2} - t), t\sqrt{k})$ returns with regard to the safe threshold of $t \leq \frac{3}{4}$ to switch from $k_{\text{found}}(t) \geq t\sqrt{k}$ to $k_{\text{found}}(t) \geq 2t(\sqrt{k+t^2} - t)$.

If $t' \geq \frac{3}{4}\sqrt{k}$, for $1 \leq t \leq \frac{3}{4}\sqrt{k}$, we are using precisely the correct bound of $t\sqrt{k}$ since thresholds smaller than t' also yield at least $\sqrt{k}$ features that contribute at least t top-k correlations. For $\frac{3}{4}\sqrt{k} \leq t \leq t'$, we are using the lower bound $2t(\sqrt{k+t^2} - t)$, which is lower than the actual lower bound of $t\sqrt{k}$. And then for $t \geq t'$, we are once again using precisely the correct

bound, i.e., $2t(\sqrt{k+t^2}-t)$. Thus, $\min(2t(\sqrt{k+t^2}-t), t\sqrt{k})$ holds for any $t' \geq \frac{3}{4}\sqrt{k}$.

Similarly, if $t' \leq \frac{3}{4}\sqrt{k}$, we can see that for $1 \leq t \leq t'$, we are using precisely the correct bound of $t\sqrt{k}$. And for $t' \leq t \leq \frac{3}{4}\sqrt{k}$, we are using the lower bound ($t\sqrt{k}$), which is lower than the actual lower bound ($2t(\sqrt{k+t^2}-t)$). And then for $t \geq \frac{3}{4}\sqrt{k}$, we are once again using precisely the correct bound of $2t(\sqrt{k+t^2}-t)$. Thus, $\min(2t(\sqrt{k+t^2}-t), t\sqrt{k})$ also holds for any $t' \leq \frac{3}{4}\sqrt{k}$.

Overall, regardless of when the switch from $T(t) \geq \sqrt{k}$ to $T(t) \leq \sqrt{k}$ happens for a given matrix, when we use $k_{\text{found}}(t) \geq \min(2t(\sqrt{k+t^2}-t), t\sqrt{k})$ we are always underestimating what is the true number of found correlations by *CorALS*. Thus, $k_{\text{found}}(t) \geq \min(2t(\sqrt{k+t^2}-t), t\sqrt{k}) = t\sqrt{k}$ for $1 \leq t \leq \frac{3}{4}\sqrt{k}$ and $k_{\text{found}}(t) \geq \min(2t(\sqrt{k+t^2}-t), t\sqrt{k}) = 2t(\sqrt{k+t^2}-t)$ for $t \geq \frac{3}{4}\sqrt{k}$. This concludes the proof of Theorem 5.3.

Corollary 5.3.1. ***Approximation factor sensitivity.*** *Based on Theorem 5.3, one can either determine the approximation factor a needed to provide a result with a minimum desired sensitivity s, or derive a minimum sensitivity s based on a given approximation factor a:*

Let the desired sensitivity be denoted as s. If the desired sensitivity is $s \leq 0.75$, then the corresponding approximation factor needs to be at least $a = s\frac{n}{\sqrt{k}}$. If the desired sensitivity s is $s \geq 0.75$, the approximation factor needs to be at least $a = \frac{sn}{2\sqrt{k}\sqrt{1-s}}$.

Equivalently, when formulating k in terms of the overall number of correlations n^2, i.e., $k = rn^2$, then for a sensitivity of $s \leq 0.75$, the approximation factor can be calculated via $a = \frac{s}{\sqrt{r}}$, and for $s \geq 0.75$ it can be calculated via $a = \frac{s}{2\sqrt{r}\sqrt{1-s}}$.

And finally, given an approximation factor a, the lower-bound sensitivity s can be estimated via $s \geq a\frac{\sqrt{k}}{n}$ ($s \geq a\sqrt{r}$) for $a \leq \frac{3}{4}\frac{n}{\sqrt{k}}$ ($a \leq \frac{3}{4\sqrt{r}}$), and $s \geq 2a(\sqrt{a^2\frac{k^2}{n^4}+\frac{k}{n^2}} - a\frac{k}{n^2})$ ($s \geq 2a(\sqrt{r^2a^2+r}-ra)$, otherwise.

Proof. If the sensitivity s desired is such that $s \leq 0.75$, Theorem 5.3 implies that choosing $t = s\sqrt{k} \leq \frac{3}{4}\sqrt{k}$ will result in at least this sensitivity. This corresponds to an approximation

factor $a = s\frac{n}{\sqrt{k}}$. If the sensitivity s desired is such that $s \geq 0.75$, Theorem 5.3 implies that choosing $t = \frac{s\sqrt{k}}{2\sqrt{1-s}}$ will result in at least this sensitivity. This corresponds to an approximation factor $a = \frac{sn}{2\sqrt{k}\sqrt{1-s}}$.

The sensitivity estimates, $s = a\frac{\sqrt{k}}{n}$ ($s = a\sqrt{r}$) for $a \leq \frac{3}{4}\frac{n}{\sqrt{k}}$, are based on a transformation of the approximation factor formula $a = s\frac{n}{\sqrt{k}}$. Similarly, for $s = 2a(\sqrt{a^2\frac{k^2}{n^4} + \frac{k}{n^2}} - a\frac{k}{n^2})$ ($s = 2a(\sqrt{r^2a^2 + r} - ar)$ in the case of $a \geq \frac{3}{4}\frac{n}{\sqrt{k}}$:

$$
\begin{aligned}
a = \frac{s \cdot n}{2\sqrt{rn^2}\sqrt{1-s}} &\Leftrightarrow s = a \cdot 2\sqrt{r}\sqrt{1-s} \\
&\Leftrightarrow s^2 = a^2 \cdot 4r(1-1) \\
&\Leftrightarrow s^2 = 4a^2r - 4a^2r1 \\
&\Leftrightarrow s^2 + 4a^2rp - 4a^2r = 0 \\
&\Leftrightarrow s = \frac{-4a^2r \pm \sqrt{16a^4r^2 + 16a^2r}}{2} \\
&\Leftrightarrow s = -2a^2r \pm 2a\sqrt{a^2r^2 + r} \\
&\Leftrightarrow s = 2a(\pm\sqrt{r^2a^2 + r} - ar)
\end{aligned}
$$

And since s is always positive, we get: $s = 2a(\sqrt{r^2a^2 + r} - ar)$.

Supplementary Section 5.2 Experimental approximation properties

Depending on the approximation factor, *CorALS* for top-k correlation network construction can return approximation results for which the previous section provided theoretical results and bounds. However, in practice the number of missed values may be substantially lower than the derived bounds suggests. To illustrate this Figure 5 shows that *CorALS* effectively produces highly accurate approximations when searching for the top-1% of correlations with approximation factors well above, e.g., the expected recall (sensitivity) of ≈ 0.83 at approximation factor $a = 10$. For this, we show precision and recall (sensitivity) with regard to which feature

pairs have been selected as top k candidates dependent on an approximation factor a. The approximation factor influences how many correlation values are inspected for each feature when calculating the overall top k correlations and thus determines how accurate the approximation will be.

Supplementary Section 6 Details on the feature representations employed by *CorALS*

Supplementary Section 6.1 Correlation projections

As mentioned in *Main Article*, "Correlation projections", the concept of *correlation projections* allows to derive a direct relationship between the correlation $cor(\boldsymbol{x}, \boldsymbol{y})$ of any two vectors and the Euclidean distance $d_e(\hat{\boldsymbol{x}}, \hat{\boldsymbol{y}})$ of their correlation projections (*32*). In particular, it holds that:

$$cor(\boldsymbol{x}, \boldsymbol{y}) = 1 - \frac{d_e(\hat{\boldsymbol{x}}, \hat{\boldsymbol{y}})^2}{2} \tag{1}$$

Proof. The Euclidean distance is defined as: $d_e(\boldsymbol{x}, \boldsymbol{y}) = \sqrt{\sum_i (x_i - y_i)^2}$. Let x, y be two features with sample vectors $\boldsymbol{x}, \boldsymbol{y}$ containing m samples and $\hat{\boldsymbol{x}}, \hat{\boldsymbol{y}}$ their respective correlation projections). Then, employing *Main Article*, "Equation 1" and the fact that $cor(\boldsymbol{z}, \boldsymbol{z}) = \langle \hat{\boldsymbol{z}}, \hat{\boldsymbol{z}} \rangle = 1$, it holds that

$$\begin{aligned}
d_e(\hat{\boldsymbol{x}}, \hat{\boldsymbol{y}}) &= \sqrt{\sum_i (\hat{x}_i - \hat{y}_i)^2} \\
&= \sqrt{\sum_i (\hat{x}_i^2 - 2\hat{x}_i y_i + \hat{y}_i^2)} \\
&= \sqrt{\sum_i \hat{x}_i^2 - \sum_i 2\hat{x}_i\hat{y}_i + \sum_i \hat{y}_i^2} \\
&= \sqrt{\langle \hat{\boldsymbol{x}}, \hat{\boldsymbol{x}} \rangle - 2\langle \hat{\boldsymbol{x}}, \hat{\boldsymbol{y}} \rangle + \langle \hat{\boldsymbol{y}}, \hat{\boldsymbol{y}} \rangle} \\
&= \sqrt{2 - 2\langle \hat{\boldsymbol{x}}, \hat{\boldsymbol{y}} \rangle} \\
&= \sqrt{2 - 2 \cdot cor(\boldsymbol{x}, \boldsymbol{y})}
\end{aligned} \tag{2}$$

And thus given the Euclidean distance $d_e(\hat{\boldsymbol{x}}, \hat{\boldsymbol{y}})$, the correlation $cor(\boldsymbol{x}, \boldsymbol{y})$ can be calculated as

$$cor(\boldsymbol{x}, \boldsymbol{y}) = 1 - \frac{d_e(\hat{\boldsymbol{x}}, \hat{\boldsymbol{y}})^2}{2}$$

Corollary: $cor(\boldsymbol{x}, \boldsymbol{y})$ and $-d_e(\hat{\boldsymbol{x}}, \hat{\boldsymbol{y}})$ are order-equivalent, i.e., for any four features x, y, p, q it holds that

$$\forall x, y, p, q : cor(\boldsymbol{x}, \boldsymbol{y}) > cor(\boldsymbol{p}, \boldsymbol{q}) \Leftrightarrow -d_e(\hat{\boldsymbol{x}}, \hat{\boldsymbol{y}}) > -d_e(\hat{\boldsymbol{p}}, \hat{\boldsymbol{q}}) \tag{3}$$

For this, note that $\sqrt{2 - 2 \cdot cor(\boldsymbol{x}, \boldsymbol{y})}$ in Equation (2) is a strictly monotone function with regard to $cor(\boldsymbol{x}, \boldsymbol{y})$ and lies in an interval of $[0, 2]$ where $d(\hat{\boldsymbol{x}}, \hat{\boldsymbol{y}}) = 0$ if $cor(\boldsymbol{x}, \boldsymbol{y}) = 1$, $d(\hat{\boldsymbol{x}}, \hat{\boldsymbol{y}}) = \sqrt{2}$ if $cor(\boldsymbol{x}, \boldsymbol{y}) = 0$, and $d(\hat{\boldsymbol{x}}, \hat{\boldsymbol{y}}) = 2$ if $cor(\boldsymbol{x}, \boldsymbol{y}) = -1$.

CorALS exploits this order-equivalence of Euclidean distance and correlation, e.g., in top correlation approximation and correlation-based embeddings.

Supplementary Section 6.2 Differential projections

Given the correlation projection $\hat{\cdot}$ introduced in *Main Article*, "Correlation projections", and the definitions in *Main Article*, "Differential projections" the subsequent proof shows that δ and κ provide a *dual vector representation* so that for any feature pair x and y sampled in two conditions (or timepoints), i.e., $\boldsymbol{x}_1, \boldsymbol{x}_2$ and $\boldsymbol{y}_1, \boldsymbol{y}_2$, it holds that

$$cor(\boldsymbol{x}_1, \boldsymbol{y}_1) - cor(\boldsymbol{x}_2, \boldsymbol{y}_2) = \langle \delta(\boldsymbol{x}_1, \boldsymbol{x}_2), \kappa(\boldsymbol{y}_1, \boldsymbol{y}_2) \rangle$$

Proof. Given *Main Article*, "Equation 1" and the correlation projection $\hat{\cdot}$ from *Main Article*, "Correlation projections", it holds that

$$cor(\boldsymbol{x}_1, \boldsymbol{y}_1) - cor(\boldsymbol{x}_2, \boldsymbol{y}_2) = \langle \hat{\boldsymbol{x}_1}, \hat{\boldsymbol{y}_1} \rangle - \langle \hat{\boldsymbol{x}_2}, \hat{\boldsymbol{y}_2} \rangle$$

Further, using the following notation for a arbitrary feature z, with m_1 and m_2 samples per feature in each condition, respecitvely,

$$\begin{aligned}\delta(\boldsymbol{z}_1, \boldsymbol{z}_2) =& (\delta_{z,1}, \ldots, \delta_{z,m_1}, \delta_{z,m_1+1}, \ldots, \delta_{z,m_1+m_2}) = (z_{1,1}, \ldots, z_{1,m_1}, z_{2,1}, \ldots, z_{2,m_2})\\ \kappa(\boldsymbol{z}_1, \boldsymbol{z}_2) =& (\kappa_{z,1}, \ldots, \kappa_{z,m_1}, \kappa_{z,m_1+1}, \ldots, \kappa_{z,m_1+m_2}) = (z_{1,1}, \ldots, z_{1,m_1}, -z_{2,1}, \ldots, -z_{2,m_2})\end{aligned}$$

it can be derived that the scalar product of the transformed vectors is equal to the correlation difference:

$$\begin{aligned}\langle \delta(\boldsymbol{x}_1, \boldsymbol{x}_2), \kappa(\boldsymbol{y}_1, \boldsymbol{y}_2)\rangle &= \sum_{i=1}^{m_1+m_2} \delta_{x,i} \cdot \kappa_{y,i}\\ &= \sum_{i=1}^{m_1} \delta_{x,i} \cdot \kappa_{y,i} + \sum_{i=m_1+1}^{m_1+m_2} \delta_{x,i} \cdot \kappa_{y,i}\\ &= \sum_{i=1}^{m_1} \hat{x}_{1,i} \cdot \hat{y}_{1,i} + \sum_{i=1}^{m_2} \hat{x}_{2,i} \cdot (-\hat{y}_{2,i})\\ &= \sum_{i=1}^{m_1} \hat{x}_{1,i} \cdot \hat{y}_{1,i} - \sum_{i=1}^{m_2} \hat{x}_{2,i} \cdot \hat{y}_{2,i}\\ &= \langle \hat{\boldsymbol{x}}_1, \hat{\boldsymbol{y}}_1\rangle - \langle \hat{\boldsymbol{x}}_2, \hat{\boldsymbol{y}}_2\rangle\\ &= cor(\boldsymbol{x}_1, \boldsymbol{y}_1) - cor(\boldsymbol{x}_2, \boldsymbol{y}_2)\end{aligned}$$

As mentioned in the main text, similar to the connection of Euclidean distance and basic correlation, the dual feature representations in the differential space exhibits a connection between Euclidean distance and correlation difference across conditions or timepoints. In particular, for two features x and y with sample vectors $\boldsymbol{x}_1$, $\boldsymbol{x}_2$ and $\boldsymbol{y}_1$, $\boldsymbol{y}_2$ across two conditions or timepoints, $cor(\boldsymbol{x}_1, \boldsymbol{y}_1) - cor(\boldsymbol{x}_2, \boldsymbol{y}_2)$ and $-d_e(\delta(\boldsymbol{x}_1, \boldsymbol{x}_2), \kappa(\boldsymbol{y}_1, \boldsymbol{y}_2))$ are order-equivalent and it holds that:

$$cor(\boldsymbol{x}_1, \boldsymbol{y}_1) - cor(\boldsymbol{x}_2, \boldsymbol{y}_2) = 2 - \frac{d_e(\delta(\boldsymbol{x}_1, \boldsymbol{x}_2), \kappa(\boldsymbol{y}_1, \boldsymbol{y}_2))^2}{2} \tag{4}$$

Proof. The proof is structurally similar to the one for correlation projections (see above): By employing *Main Article*, "Equation 1" and the fact that $cor(\boldsymbol{z}, \boldsymbol{z}) = \langle \hat{\boldsymbol{z}}, \hat{\boldsymbol{z}} \rangle = 1$, it holds that

$$
\begin{aligned}
& d_e(\delta(\boldsymbol{x}_1, \boldsymbol{x}_2), \kappa(\boldsymbol{y}_1, \boldsymbol{y}_2)) = \\
&= \sqrt{\sum_{i=1}^{m_1+m_2} (\delta_{x,i} - \kappa_{y,i})^2} \\
&= \sqrt{\sum_{i=1}^{m_1+m_2} (\delta_{x,i}^2 - 2\delta_{x,i}\kappa_{y,i} + \kappa_{y,i}^2)} \\
&= \sqrt{\sum_{i=1}^{m_1+m_2} \delta_{x,i}^2 - \sum_{i=1}^{m_1+m_2} 2\delta_{x,i}\kappa_{y,i} + \sum_{i=1}^{m_1+m_2} \kappa_{y,i}^2} \\
&= \sqrt{\sum_{i=1}^{m_1} \hat{x}_{1,i}^2 + \sum_{i=m_1+1}^{m_1+m_2} \hat{x}_{2,i}^2 - 2\left(\sum_{i=1}^{m_1} \hat{x}_{1,i}\hat{y}_{1,i} + \sum_{i=m_1+1}^{m_1+m_2} \hat{x}_{2,i}(-\hat{y}_{2,i})\right) + \sum_{i=1}^{m_1} \hat{y}_{1,i}^2 + \sum_{i=m_1+1}^{m_1+m_2} (-\hat{y}_{2,j})^2} \\
&= \sqrt{\langle \hat{\boldsymbol{x}_1}, \hat{\boldsymbol{x}_1} \rangle + \langle \hat{\boldsymbol{x}_2}, \hat{\boldsymbol{x}_2} \rangle - 2(\langle \hat{\boldsymbol{x}_1}, \hat{\boldsymbol{y}_1} \rangle - \langle \hat{\boldsymbol{x}_2}, \hat{\boldsymbol{y}_2} \rangle) + \langle \hat{\boldsymbol{y}}, \hat{\boldsymbol{y}} \rangle + \langle \hat{\boldsymbol{y}_2}, \hat{\boldsymbol{y}_2} \rangle} \\
&= \sqrt{4 - 2(\langle \hat{\boldsymbol{x}_1}, \hat{\boldsymbol{y}_1} \rangle - \langle \hat{\boldsymbol{x}_2}, \hat{\boldsymbol{y}_2} \rangle)} \\
&= \sqrt{4 - 2(cor(\boldsymbol{x}_1, \boldsymbol{y}_1) - cor(\boldsymbol{x}_2, \boldsymbol{y}_2))}
\end{aligned}
\tag{5}
$$

And thus given the Euclidean distance $d_e(\delta(\boldsymbol{x}_1, \boldsymbol{x}_2), \kappa(\boldsymbol{y}_1, \boldsymbol{y}_2))$, the correlation difference $cor(\boldsymbol{x}_1, \boldsymbol{y}_1) - cor(\boldsymbol{x}_2, \boldsymbol{y}_2)$ can be calculated as

$$cor(\boldsymbol{x}_1, \boldsymbol{y}_1) - cor(\boldsymbol{x}_2, \boldsymbol{y}_2) = 2 - \frac{d_e(\delta(\boldsymbol{x}_1, \boldsymbol{x}_2), \kappa(\boldsymbol{y}_1, \boldsymbol{y}_2))^2}{2}$$

Corollary: $cor(\boldsymbol{x}_1, \boldsymbol{y}_1) - cor(\boldsymbol{x}_2, \boldsymbol{y}_2)$ and $-d_e(\delta(\boldsymbol{x}_1, \boldsymbol{x}_2), \kappa(\boldsymbol{y}_1, \boldsymbol{y}_2))$ are order-equivalent, i.e.,

$$
\begin{aligned}
& \forall x, y, p, q : \\
& cor(\boldsymbol{x}_1, \boldsymbol{y}_1) - cor(\boldsymbol{x}_2, \boldsymbol{y}_2) > cor(\boldsymbol{p}_1, \boldsymbol{q}_1) - cor(\boldsymbol{p}_2, \boldsymbol{q}_2) \\
& \Leftrightarrow -d_e(\delta(\boldsymbol{x}_1, \boldsymbol{x}_2), \kappa(\boldsymbol{y}_1, \boldsymbol{y}_2)) > -d_e(\delta(\boldsymbol{p}_1, \boldsymbol{p}_2), \kappa(\boldsymbol{q}_1, \boldsymbol{q}_2))
\end{aligned}
\tag{6}
$$

For this, note that $\sqrt{4-2(cor(\boldsymbol{x}_1,\boldsymbol{y}_1)-cor(\boldsymbol{x}_2,\boldsymbol{y}_2))}$ in Equation (5) is a strictly monotone function with regard to $cor(\boldsymbol{x}_1,\boldsymbol{y}_1)-cor(\boldsymbol{x}_2,\boldsymbol{y}_2)$ and lies in an interval of $[0,\sqrt{8}]$, where for example

$$\begin{aligned}
d_e(\delta(\boldsymbol{x}_1,\boldsymbol{x}_2),\kappa(\boldsymbol{y}_1,\boldsymbol{y}_2)) &= 0 \text{ if } cor(\boldsymbol{x}_1,\boldsymbol{y}_1)=1 \wedge cor(\boldsymbol{x}_2,\boldsymbol{y}_2)=-1\\
d_e(\delta(\boldsymbol{x}_1,\boldsymbol{x}_2),\kappa(\boldsymbol{y}_1,\boldsymbol{y}_2)) &= \sqrt{8} \text{ if } cor(\boldsymbol{x}_1,\boldsymbol{y}_1)=-1 \wedge cor(\boldsymbol{x}_2,\boldsymbol{y}_2)=1\\
d_e(\delta(\boldsymbol{x}_1,\boldsymbol{x}_2),\kappa(\boldsymbol{y}_1,\boldsymbol{y}_2)) &< \sqrt{4} \text{ if } cor(\boldsymbol{x}_1,\boldsymbol{y}_1)-cor(\boldsymbol{x}_2,\boldsymbol{y}_2)<0\\
d_e(\delta(\boldsymbol{x}_1,\boldsymbol{x}_2),\kappa(\boldsymbol{y}_1,\boldsymbol{y}_2)) &> \sqrt{4} \text{ if } cor(\boldsymbol{x}_1,\boldsymbol{y}_1)-cor(\boldsymbol{x}_2,\boldsymbol{y}_2)>0
\end{aligned}$$

Analogously to correlation projections, *CorALS* exploits this order-equivalence of Euclidean distance and correlation for top differential correlation approximation.

Supplementary Section 7 Performance of correlation embeddings

Some t-SNE implementations support pre-computed distance matrices or custom distance functions which can be used to provide correlation-based distance information; however this is often inefficient. For example, calculating standard feature embeddings for the relatively small pregnancy dataset (see *Main Article*, ”Table 1”) with Scikit-learn's (*33*) t-SNE implementation requires $\sim$ 500 MB of memory and takes $\sim$ 10 minutes. However, providing a pre-computed distance matrix, which can be used to incorporate correlation information, increases memory consumption to $\sim$ 16 GB. Alternatively, using a corresponding custom distance metric increases runtimes from $\sim$ 10 minutes to several hours making this approach infeasible. In contrast, by projecting the features onto correlation vectors, *CorALS* establishes an order equivalence between Euclidean distance and correlation as introduced in *Main Article*, ”Correlation embeddings”. This allows to directly employ distance-based embeddings methods like t-SNE on the projected features without adding substantial computational overhead or requiring implemen-

tations that support customized distance information.

Supplementary Section 8 Addendum to ”Large-scale multiomics correlation analysis across pregnancy”

As mentioned in *Main Article*, ”Large-scale multiomics correlation analysis across pregnancy”, this experiment demonstrates *CorALS*’s potential in multiomics studies by analyzing feature correlations in a dataset containing 3rd trimester and postpartum biospecimens from healthy pregnant women. The following paragraphs represent a more detailed and extended version of the analysis in the main text.

As previously described in the main text, a particularly striking feature in *Main Article*, ”Figure 2” is the batch of correlation edges between the transcriptome (cell-free RNA) and the immunome that appears in the third trimester but vanishes postpartum. A closer examination of the relevant features reveals a module of genes positively correlated with an intracellular signaling response - p38 phosphorylation - across several immune cell subsets. P38-family proteins are mitogen-activated protein kinases (MAPKs) specifically induced by stimuli such as oxidative stress and inflammation. P38 are essential for both innate immunity via the response to endotoxin and in adaptive immunity via the mediation of T-cell activation (*34*). During gestation, oxidative stress arises from maternal adaptations to fetal growth. There is also evidence that p38 plays a role in the regulation of pregnancy and parturition, but its exact mechanism is still poorly understood (*35*). On the other hand, the cell-free RNA features (e.g., RFXANK,ZNF831,SH3BP5,150ICA1, and GATAD1) with the most associations (i.e. edges) with p38 phosphorylation have previously been reported to be associated with immune function or pregnancy. Regulatory Factor X Associated Ankyrin Containing Protein (*RFXANK*) is a known transcriptional regulator of certain MHCII genes, which are responsible for antigen presentation in adaptive immunity (*36*). Zinc Finger Protein 831 (*ZNF831*) is involved in the adaptive im-

mune response and has been linked to preeclampsia, an inflammatory and hypertensive disorder of pregnancy (*37, 38*). SH3 Domain Binding Protein 5 (*SH3BP5*) and Islet Cell Autoantigen 1 (*ICA1*) have also been associated with preeclampsia via transcriptional and epigenetic mechanisms respectively (*39, 40*). Finally, GATA Zinc Finger Domain Containing 1 (*GATAD1*) is a transcription factor downregulated in preeclamptic placentas via epigenetic processes (*41*). Gene ontology enrichment analysis of the set of genes correlated with at least one mass cytometry pP38-related feature in the third trimester showed an enrichment of pathways related to chromatin remodeling, including histone acetylation, chromatin silencing, and chromatin organization. While no definite conclusions can be drawn, these results can be used for biological hypothesis generation. Specifically, they highlight the potential of exploring the mechanistic role of p38 in pregnancy and also offer multiple candidate genes which might be involved in this process.

Another set of note-worthy edges with large correlation differences from the third trimester to postpartum is the batch of edges between the cell-free RNA modality and the microbiome. A woman's vaginal, oral, and gut microbial landscapes play key roles in the healthy progression of pregnancy, specifically through nutrient metabolism and immune regulation (*42*). Further interrogation of this batch of edges reveals a module of genes (e.g., KYNU, ZC3H12D, and MAP3K14) with previously characterized connections to the microbiome and pregnancy in the literature. Kynureninase (*KYNU*) plays a role in tryptophan biosynthesis, through which it has been associated with the crosstalk between the host and the microbiome in the gut and across multiple dermal pathologies (*43–45*). Moreover, *KYNU* is essential for proper embryonic development and gestation, outlining a connection between the microbiome and a healthy pregnancy (*46*). Zinc Finger CCCH-Type Containing 12D (*ZC3H12D*) is a negative regulator of toll-like receptor signaling and inflammation (*47, 48*) which has been found to be differentially methylated during pregnancy and particularly in the cord blood of pregnancies affected by

prematurity or a hypertensive disorder (*49, 50*). Furthermore, there is evidence of regulation of the methylation of *ZC3H12D* by gut microbiota (*51*). Mitogen-Activated Protein Kinase Kinase Kinase 14 (*MAP3K14*) is a crucial activator of the non-canonical NF-kB pathway, a signaling pathway implicated in placental function and the duration of pregnancy (*52, 53*). *MAP3K14* has also been shown to be essential in the maintenance of gut microbial homeostasis through its activity in dendritic cells (*54*). Other relevant genes in this set included GIPC PDZ Domain Containing Family Member 1 (*GIPC1*), Complement C1q B Chain (*C1QB*), and Basic Leucine Zipper ATF-Like Transcription Factor 2 (*BATF2*), which have all been associated with the microbiome across multiple tissues (*55–59*). Altogether, this batch of edges between the cell-free RNA modality and the microbiome modality highlight the different ways the maternal microbial landscape can directly impact the progression of pregnancy through interactions with immune and metabolic biological processes.

Similarly, there are various smaller batches of correlations between cell-free RNA and the protein-related modalities (plasma and serum based proteome measurements) that change substantially between the two timepoints. Some of these include correlations involving immune cytokines like IFN-γ, IL-10, and IL-1RA, metabolic proteins like GPD1, and growth factors like PDGFBB.

Supplementary Section 9 Addendum to ”Correlated functional changes across immune cells”

As mentioned in *Main Article*, ”Correlated functional changes across immune cells”, *CorALS* enables the analysis the coordination of individual cells across concerted immune responses based on their functional correlation. Particularly, *Main Article*, ”Figure 3” visualizes the amount and direction of change in the relative number of functional cell correlations attributed to individual cell type pairs within the top-k functional cell correlations between the third

trimester and postpartum. These changes mostly revolve around B cells and CD56^{dim}CD16^{+} NK cells. While a detailed analysis may be of interest, we focus on these changes as an example in order to illustrate the complementary perspectives enabled by *CorALS*.

CD56^{dim}CD16^{+} NK cell correlation changes. Peripheral NK cells are connected to important processes during pregnancy. For example, peripheral CD56^{dim}CD16^{+} NK cells have been shown to promote tolerance early in pregnancy (*60*), and there is evidence of increased activation of this cell subset during the second and third trimesters, possibly due to increased pro-inflammatory stimuli from monocytes and dendritic cells (*61*). In line with this, *Main Article*, ”Figure 3” indicates that CD56^{dim}CD16^{+} NK cells in the third trimester functionally align to classical and intermediate monocytes, which have also been previously described to activate during late pregnancy (*62, 63*). While further work would be needed to determine the mechanism underpinning these changes in correlations, pregnancy-related hormones have been demonstrated to modulate the function of both NK cells and monocytes (*64, 65*), pointing towards a candidate source of the observed immune orchestration.

B cell correlation changes. The role of B cells during pregnancy has only recently come into focus. Particularly, studies have highlighted their immunosuppressive potential in maintaining maternal-fetal tolerance and how the dysregulation of immunosuppressive B cells can lead to adverse pregnancy outcomes (*66, 67*). Previous work reported altered levels of B cell activation markers in the serum of pregnant women in the third trimester when compared to the postpartum period and to healthy controls (*68*). Along these lines, the changes in correlations observed in *Main Article*, ”Figure 3” suggest that, in the third trimester, B cells increase in signaling response similarity to innate immunosuppressive cell subsets such as the monocytic myeloid-derived suppressor cells (M-MDSCs). This may be a response to control systemic inflammation and prevent early parturition or a result of the altered B-cell marker profile in the blood reported to occur at the end of pregnancy.

The previous observations suggest that B cells and CD56^{dim}CD16^{+} NK cells acquire an intracellular signaling signature in the third trimester that overlaps with functional signatures of innate immune cells, as suggested by the increased relative number of correlations between B cells and M-MDSCs and between CD56^{dim}CD16^{+} NK cells and classical as well as intermediate monocytes. Postpartum, these two cell types and various T cell subsets shift functionally to more similar signalling response signatures, suggesting a return to their pre-pregnancy state and the postpartum release of the pregnancy-associated Th2 polarization of the T cell compartment.

Supplementary Section 10 A brief history on the correlation coefficient

The history of the correlation coefficient goes back over one hundred years, with most of its theoretical evolution before 1920. Incidentally, last year 2020, marked the 100th anniversary since the celebrated Karl Pearson article "Notes on the history of correlation" was published in the journal *Biometrica*. In this historical overview article (*69*), Pearson tried to connect all the factors that contributed to the development of the correlation coefficient from different scientists. He argued that to understand the assertions of correlational calculus, one must go back to Gauss's fundamental memoirs on least squares. In fact, and this might come as a surprise to the reader since the concept of correlation often appears before regression in Statistics books, the concept of regression and least squares preceded the concept of correlation historically (*70*). One of the commonly known first insights on regression (also known as reversion) came from Sir Francis Galton when he was interested in comparing the sizes of daughter peas against the sizes of mother peas (*70*). Pearson concluded his article by saying that the paper was a "long step from Francis Galton's "reversion" in sweet pea seeds to the full theory of multiple correlation."

There are many diverse ways to view the correlation coefficient (we use the terms Pearson's correlation coefficient and correlation coefficient interchangeably in this paper), and the authors

of (*71*) discuss thirteen of them. Perhaps the most common way to think about the correlation coefficient is to go back to the proposed statement by Karl Pearson in 1895, named the Pearson product-moment correlation coefficient. We provide a more algebraic variant of the original formula from Pearson's 1895 paper in Equation 7.

$$r = \frac{\sum_i (\boldsymbol{x}_i - \mu_{\boldsymbol{x}})(\boldsymbol{y}_i - \mu_{\boldsymbol{y}})}{\sqrt{\sum_i (\boldsymbol{x}_i - \mu_{\boldsymbol{x}})^2 \sum_i (\boldsymbol{x}_i - \mu_{\boldsymbol{x}})^2}} \tag{7}$$

Here, μ_i represents the mean across all samples for a given variable i. To incentivize the intuition behind this formula, we can go back to one of the earliest examples on the topic: sweet peas. What Galton wanted to understand was the relationship between the size of a parent sweet pea and a child sweet pea by collecting many measurements from different parents and children. A key component to note here is that Galton was not interested in a generational difference, i.e. he did not want to compare children pea sizes to parents pea sizes, and instead, wanted to compare multiple parent-child sizes pairs to each other. This is crucial to see, as it can help us realize what the formula is trying to measure for two variables—how two variables change together while not biasing the relation by the rate of change. In the numerator, this is achieved by centering both vectors by subtracting out the mean of each variable, and the denominator scales both vectors to have equal units. For any two vectors, the correlation value r is between -1 and $+1$ and this can be shown by using the Cauchy-Schwarz inequality (*71*).

Arguably, the idea of correlation made its earliest appearance in biology but it quickly transcended to other fields. In an article published by Galton in 1889, he states "Correlation of structure is a phrase much used in biology, and not least in that branch of it which refers to heredity" (*72*). Prior to that, Galton's cousin, Charles Darwin published his book "The Variation of Animals and Plants Under Domestication" in 1868 and used the concept of correlation multiple times (*73*). Since then, the concept of correlation has made it to a diverse set of fields such as physics, astronomy, chemistry, psychology, and more. In these fields, and many oth-

ers, the notion of correlation matrices emerged—i.e. matrices where every entry represents a correlation coefficient between a pair of features. The wide presence of correlation matrices in multiple fields can be justified by realizing the predictive power that correlations have. For instance, realizing a strong correlation between a given disease severity and the presence of a certain level of a protein in the body can help us understand the disease and help guide the drug manufacturing process. And, in fact, the concept of correlations analysis has survived the test of time in biology and is widely used. At the time of writing this manuscript, we found that a search of the term "correlation" on bioRxiv revealed 63,942 articles out of the 94,234 articles present at the time. This is 67% of all articles on bioRxiv!

Part II
Supplementary Algorithms

Supplementary Algorithm 1: Top k correlation network approximation

Input : $X \in \mathbb{R}^{m \times n}$ (sample-vector matrix),
k (number of top correlations to retrieve),
a (approximation factor)

Output: C (approximation of top k correlations)
F' (feature pairs F' corresponding to C)

// initialize
$k' := a \cdot \left\lceil \frac{k}{n} \right\rceil$
$\hat{X}$:= preprocess each column $\hat{\boldsymbol{x}}$ so that $\hat{\boldsymbol{x}} = \frac{\boldsymbol{x} - \mu_{\boldsymbol{x}}}{\|\boldsymbol{x} - \mu_{\boldsymbol{x}}\|}$ (see *Main Article*, ”Equation 1”)
$T := build\_tree(\hat{X} \cup -\hat{X})$

// for each query feature in $\hat{X}$, search tree T for k' nearest neighbors
$D, F := search(T, \hat{X}, k')$

// merge results from individual queries into a global top k estimate
$D', F' := merge(D, F)$

// convert Euclidean distance to correlation values (for each $d \in D$)
$C := 1 - \frac{D^2}{2}$ (see *Main Article*, ”Equation 2”)

Part III
Supplementary Figures and Tables

Supplementary figures and tables are listed on the following pages.

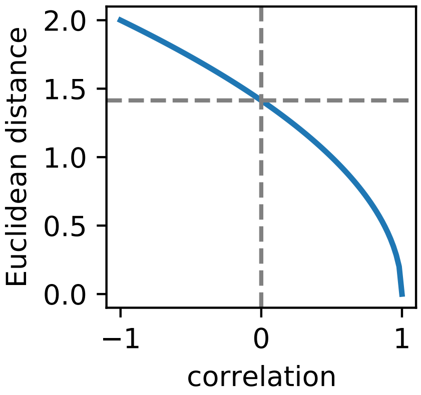

Supplementary Figure 1: **Illustration of the relationship between feature correlations and Euclidean distance after applying *CorALS*'s correlation projection.** Corresponding sample vectors were transformed using *CorALS*'s correlation projection scheme and their correlations and Euclidean distances calculated.

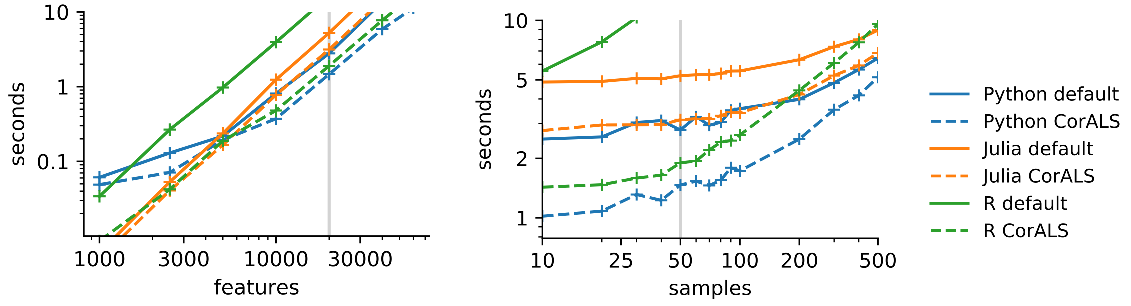

(a) Varying features

(b) Varying samples

Supplementary Figure 2: **Full correlation matrix - Runtimes on synthetic data.** Runtime comparison of *CorALS* (in seconds) for calculating the full correlation matrix on synthetic data, respectively, for the programming languages Julia, Python, and R, using a single thread. The *CorALS* implementations substantially outperform traditional correlation functions.

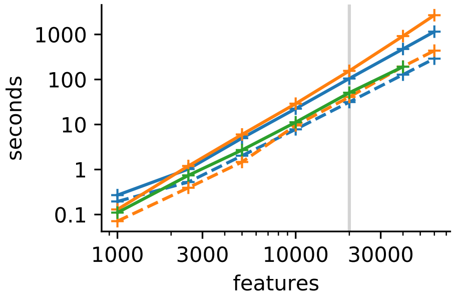

(a) Varying features

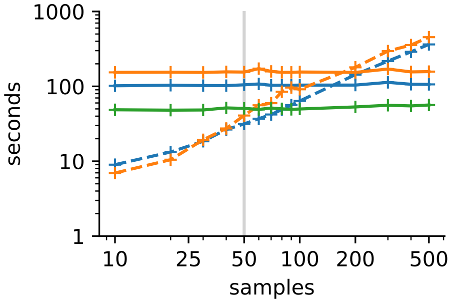

(b) Varying samples

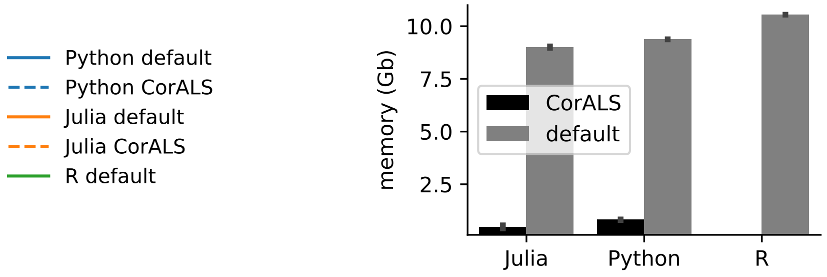

(c) Memory consumption on 50 samples and 20,000 features

Supplementary Figure 3: **Top k correlation network approximation - Runtimes and memory consumption on synthetic data.** Subfigures (a) and (b) show timing comparisons between sorting based (default) and *CorALS*'s tree-based (*CorALS*) approaches on synthetic data for the programming languages Julia, Python, and R using a single thread. (c) visualizes the corresponding memory consumption including minimal error bars. For the individual bars, the measure of center is the arithmetic mean with error bars representing the confidence interval of CI=0.95; individual data points are too dense to visualize.

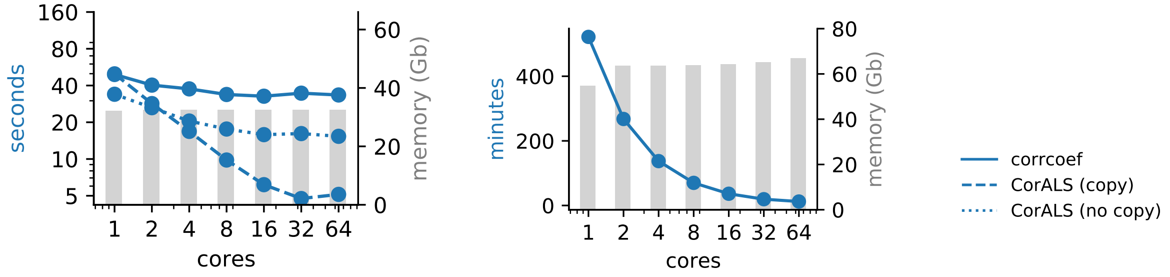

(a) Full correlation

(b) Top-k

Supplementary Figure 4: **Parallelization experiments for full and top-k correlation computation.** Runtime and memory comparisons of *CorALS* on synthetic data as a function of the number of cores used. Blue lines refer to runtime, grey bars refer to memory consumption. Both implementations (full correlation and top-k) are inherently parallelizable. For full correlation matrix computation it can be important to provide a copy of the original sample-feature matrix as more cores are utilized to gain the most speedup (see *copy* vs *no copy*).

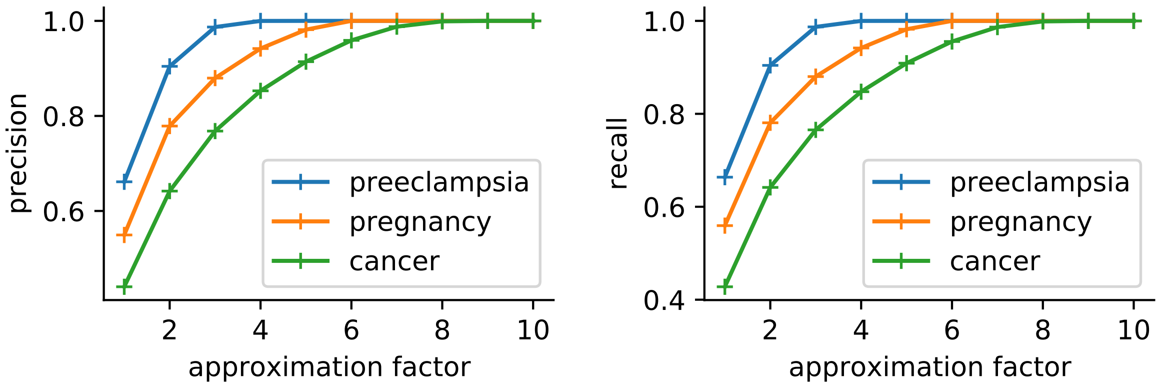

(a) Precision

(b) Recall

Supplementary Figure 5: **Top-k correlation approximation - Quality.** Precision and recall (sensitivity) of *CorALS*'s top-k approximation approach in the preeclampsia, pregnancy, and cancer datasets as a function of the approximation factor used.

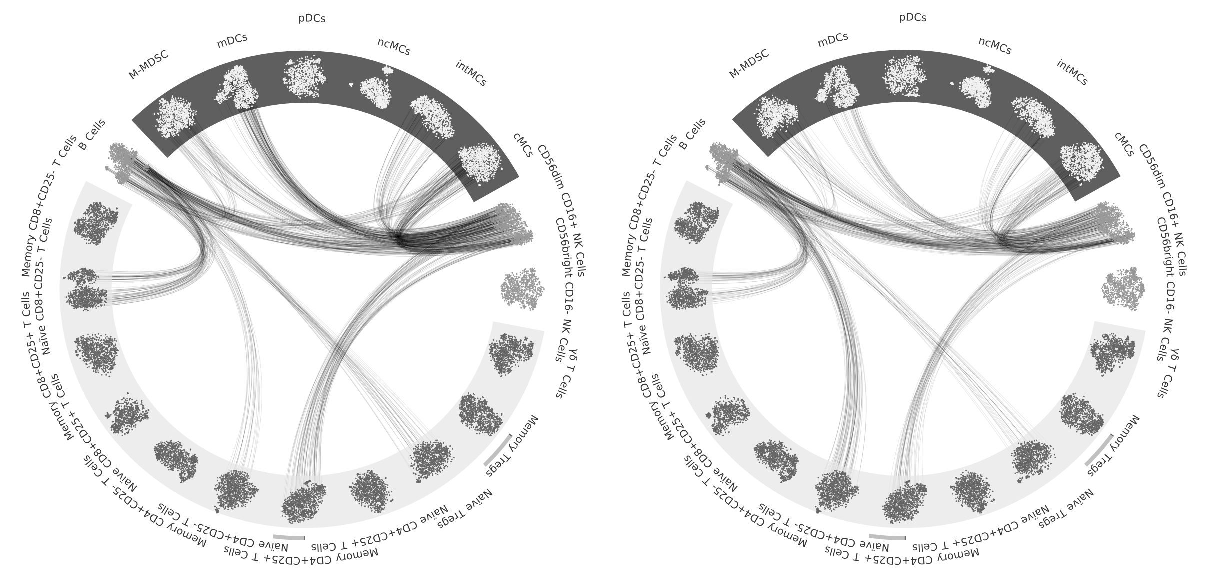

(a) Third trimester

(b) Postpartum

Supplementary Figure 6: **Individual top-k functional correlations of single cells for the third trimester of pregnancy and postpartum.** Both panels visualize cells arranged by cell types (scatter plots along the circle) and their overall top-k (k=0.01%) functional correlations (edges) for a single sample of our bootstrapping procedure used in *Main Article*, ”Large-scale multi-omics correlation analysis across pregnancy”. For visualization purposes, the number of cells per cell type is limited to 1,000, and edges are limited to cell type pairs that exhibit *very large* effect sizes (Cliff’s δ, threshold $t = 0.622$) with regard to their difference in the relative number of top-k correlations across the third trimester and postpartum. The scatter plots of single cells for each cell type are visualized using *CorALS*’s correlation-based t-SNE embeddings.

	twice	no-dual	joint
Preeclampsia	16.6	18.7	14.3
Pregnancy	1:57.1	4:34.1	1:49.2
Cancer (0.25)	33:07.0	55:58.8	32:54.4
Preeclampsia	1.0 GB	0.7 GB	0.7 GB
Pregnancy	2.4 GB	1.3 GB	1.3 GB
Cancer (0.25)	8.7 GB	4.8 GB	4.1 GB

Supplementary Table 1: **Full correlation matrix - Runtime and memory for different top-k search techniques.** The runtime is shown in the top and memory bottom half of the table. *twice* refers to running the top k search twice for extracting positive and negative correlations, while *joint* refers to jointly building a ball tree based on positive and negative features. The latter has marginal runtime advantages and reduces memory requirements by half.

	cor	WGCNA	coop	Rfast	HiClimR
Preeclampsia	7.9	2.8	1.7	2.3	10.2
Pregnancy	1:16.5	10.1	6.9	9.0	1:28.7
Cancer (0.25)	19:40.3	1:37.2	failed	1:11.5	19:57.0
Cancer (0.50)	1:33:24.5	10:51.3	failed	9:11.9	-
Cancer (1.00)	-	-	-	-	-
Single Cell	-	-	-	-	-
Preeclampsia	2.1 GB	2.1 GB	2.1 GB	2.1 GB	6.4 GB
Pregnancy	7.7 GB	7.8 GB	7.7 GB	7.8 GB	23.3 GB
Cancer (0.25)	31.2 GB	31.3 GB	-	31.5 GB	94.1 GB
Cancer (0.50)	124.7 GB	125.0 GB	-	125.4 GB	-
Cancer (1.00)	-	-	-	-	-
Single Cell	-	-	-	-	-

	CorALS	***CorALS* (top-k)**	***CorALS* (top-k, parallel)**
Preeclampsia	1.0	14.3	2.4
Pregnancy	3.9	1:49.2	5.7
Cancer (0.25)	33.9	32:54.4	59.6
Cancer (0.50)	2:20.0	2:10:25.2	2:58.4
Cancer (1.00)	-	8:42:12.9	11:25.3
Single Cell	-	16:10.1	1:46.9
Preeclampsia	2.5 GB	0.7 GB	3.4 GB
Pregnancy	8.2 GB	1.3 GB	4.5 GB
Cancer (0.25)	31.5 GB	4.1 GB	8.7 GB
Cancer (0.50)	125.9 GB	14.3 GB	21.1 GB
Cancer (1.00)	-	53.5 GB	65.1 GB
Single Cell	-	33.2 GB	38.7 GB

Supplementary Table 2: **Runtime comparison of various libraries for efficient correlation matrix calculation.** Runtimes are reported in `(hours:)minutes:seconds` (top half of the table) and memory consumption is reported in gigabytes (GB). None of the compared methods uses only native code as *CorALS* does. `coop` fails on large datasets (*failed*). Dashes (-) represent the lack of runtime measurements for examples exceeding our server resources. The *CorALS* reference implementation in Python outperforms all compared libraries on the given tasks. We also include *CorALS*'s top-k (k=0.01%) correlation extraction for direct comparison. This illustrates the ability of *CorALS* to enable correlation analysis for large scale datasets in settings with limited memory (note that is also sorts and extracts the top-k correlations which non top-k variants do not inherently support).

Short Name	Long Name
mDCs	Myeloid Dendritic Cells
pDCs	Plasmacytoid Dendritic Cell
ncMCs	Non-classical Monocytes
intMCs	Intermediate Monocytes
cMCs	Classical Monocytes
M-MDSC	Monocytic Myeloid-Derived Suppressor Cells
B Cells	B Cells
Memory CD8+CD25- T Cells	Memory CD8+CD25- T Cells
Naïve CD8+CD25- T Cells	Naïve CD8+CD25- T Cells
Memory CD8+CD25+ T Cells	Memory CD8+CD25+ T Cells
Naïve CD8+CD25+ T Cells	Naïve CD8+CD25+ T Cells
Memory CD4+CD25- T Cells	Memory CD4+CD25- T Cells
Naïve CD4+CD25- T Cells	Naïve CD4+CD25- T Cells
Memory CD4+CD25+ T Cells	Memory CD4+CD25+Foxp3- T Cells
Naïve CD4+CD25+ T Cells	Naïve CD4+CD25+Foxp3- T Cells
Naïve Tregs	Naïve Regulatory T Cells
Memory Tregs	Memory Regulatory T Cells
$\gamma\delta$ T Cells	$\gamma\delta$ T Cells
CD56bright CD16- NK Cells	CD56bright CD16- NK Cells
CD56dim CD16+ NK Cells	CD56dim CD16+ NK Cells

Supplementary Table 3: **Cell type name abbreviations.** The left columns (short name) defines a more concise naming scheme for the cell types listed in the right column (long name).

Supplementary Section 10 References

1. Andoni, A. & Indyk, P. *Near-optimal hashing algorithms for approximate nearest neighbor in high dimensions*, 459–468 (IEEE, 2006).

2. Curtin, R. *et al.* *Tree-independent dual-tree algorithms*, 1435–1443 (2013). Note.

3. Langfelder, P. & Horvath, S. Fast R functions for robust correlations and hierarchical clustering. *Journal of Statistical Software* **46** (11), 1–17 (2012). URL `https://www.jstatsoft.org/v46/i11/`.

4. Papadakis, M. *et al.* *Rfast: A Collection of Efficient and Extremely Fast R Functions* (2021). URL `https://CRAN.R-project.org/package=Rfast`. R package version 2.0.3.

5. Schmidt, D. Co-Operation: Fast correlation, covariance, and cosine similarity (2021). URL `https://cran.r-project.org/package=coop`. R package version 0.6-3.

6. Badr, H. S., Zaitchik, B. F. & Dezfuli, A. K. A tool for hierarchical climate regionalization. *Earth Science Informatics* **8** (4), 949–958 (2015). URL `https://doi.org/10.1007/s12145-015-0221-7`. `https://doi.org/10.1007/s12145-015-0221-7`.

7. Traxl, D., Boers, N. & Kurths, J. Deep graphs—a general framework to represent and analyze heterogeneous complex systems across scales. *Chaos: An Interdisciplinary Journal of Nonlinear Science* **26** (6), 065303 (2016) .

8. Rocklin, M. *Dask: Parallel computation with blocked algorithms and task scheduling*, 123–132 (Citeseer, 2015).

9. Zaharia, M., Chowdhury, M., Franklin, M. J., Shenker, S. & Stoica, I. *Spark: Cluster computing with working sets* (2010).

10. Okuta, R., Unno, Y., Nishino, D., Hido, S. & Loomis, C. *Cupy: A numpy-compatible library for nvidia gpu calculations* (2017). URL `http://learningsys.org/nips17/assets/papers/paper_16.pdf`.

11. Raschka, S., Patterson, J. & Nolet, C. Machine learning in python: Main developments and technology trends in data science, machine learning, and artificial intelligence. *Information* **11** (4), 193 (2020) .

12. Eslami, T. & Saeed, F. Fast-gpu-pcc: A gpu-based technique to compute pairwise pearson's correlation coefficients for time series data—fmri study. *High-throughput* **7** (2), 11 (2018) .

13. Chang, D.-J., Desoky, A. H., Ouyang, M. & Rouchka, E. C. *Compute pairwise manhattan distance and pearson correlation coefficient of data points with gpu*, 501–506 (IEEE, 2009).

14. Kijsipongse, E., Suriya, U., Ngamphiw, C., Tongsima, S. *et al.* *Efficient large pearson correlation matrix computing using hybrid mpi/cuda*, 237–241 (IEEE, 2011).

15. Wang, S. *et al.* Optimising parallel r correlation matrix calculations on gene expression data using mapreduce. *BMC bioinformatics* **15** (1), 1–9 (2014) .

16. Chilson, J., Ng, R., Wagner, A. & Zamar, R. Parallel computation of high-dimensional robust correlation and covariance matrices. *Algorithmica* **45** (3), 403–431 (2006) .

17. Kim, S. ppcor: an r package for a fast calculation to semi-partial correlation coefficients. *Communications for statistical applications and methods* **22** (6), 665 (2015) .

18. Xiong, H., Brodie, M. & Ma, S. *Top-cop: Mining top-k strongly correlated pairs in large databases*, 1162–1166 (IEEE, 2006).

19. McKenzie, A. T., Katsyv, I., Song, W.-M., Wang, M. & Zhang, B. Dgca: a comprehensive r package for differential gene correlation analysis. *BMC systems biology* **10** (1), 106 (2016) .

20. Jardim, V. C., Santos, S. d. S., Fujita, A. & Buckeridge, M. S. Bionetstat: a tool for biological networks differential analysis. *Frontiers in genetics* 594 (2019) .

21. Tu, J.-J. *et al.* Differential network analysis by simultaneously considering changes in gene interactions and gene expression. *Bioinformatics* **37** (23), 4414–4423 (2021) .

22. Ha, M. J., Baladandayuthapani, V. & Do, K.-A. Dingo: differential network analysis in genomics. *Bioinformatics* **31** (21), 3413–3420 (2015) .

23. Fukushima, A. Diffcorr: an r package to analyze and visualize differential correlations in biological networks. *Gene* **518** (1), 209–214 (2013) .

24. Siska, C., Bowler, R. & Kechris, K. The discordant method: a novel approach for differential correlation. *Bioinformatics* **32** (5), 690–696 (2016) .

25. Ghazanfar, S., Strbenac, D., Ormerod, J. T., Yang, J. Y. & Patrick, E. Dcars: differential correlation across ranked samples. *Bioinformatics* **35** (5), 823–829 (2019) .

26. Anderson, E. *et al.* *LAPACK Users' Guide* Third edn (Society for Industrial and Applied Mathematics, Philadelphia, PA, 1999).

27. Blackford, L. S. *et al.* An updated set of basic linear algebra subprograms (blas). *ACM Transactions on Mathematical Software* **28** (2), 135–151 (2002) .

28. Xiang, W. *Analysis of the time complexity of quick sort algorithm*, Vol. 1, 408–410 (IEEE, 2011).

29. Musser, D. R. Introspective sorting and selection algorithms. *Software: Practice and Experience* **27** (8), 983–993 (1997) .

30. Omohundro, S. M. Five balltree construction algorithms. Tech. Rep. TR-89-063, International Computer Science Institute (1989).

31. Cislak, A. & Grabowski, S. *Experimental evaluation of selected tree structures for exact and approximate k-nearest neighbor classification*, 93–100 (IEEE, 2014).

32. Greenacre, M. & Primicerio, R. *Multivariate analysis of ecological data* (Fundacion BBVA, 2014).

33. Pedregosa, F. *et al.* Scikit-learn: Machine learning in Python. *Journal of Machine Learning Research* **12**, 2825–2830 (2011) .

34. Ashwell, J. D. The many paths to p38 mitogen-activated protein kinase activation in the immune system. *Nature Reviews Immunology* **6** (7), 532–540 (2006). URL `https://doi.org/10.1038/nri1865`. `https://doi.org/10.1038/nri1865` .

35. Sheller-Miller, S., Richardson, L., Martin, L., Jin, J. & Menon, R. Systematic review of p38 mitogen-activated kinase and its functional role in reproductive tissues. *American Journal of Reproductive Immunology* **80** (6), e13047 (2018). URL `https://doi.org/10.1111/aji.13047`. `https://doi.org/10.1111/aji.13047`, publisher: John Wiley & Sons, Ltd .

36. Masternak, K. *et al.* A gene encoding a novel RFX-associated transactivator is mutated in the majority of MHC class II deficiency patients. *Nature Genetics* **20** (3), 273–

277 (1998). URL https://doi.org/10.1038/3081. https://doi.org/10.1038/3081.

37. da Silveira, W. A. *et al.* Transcription Factor Networks derived from Breast Cancer Stem Cells control the immune response in the Basal subtype. *Scientific Reports* **7** (1), 2851 (2017). URL https://doi.org/10.1038/s41598-017-02761-6. https://doi.org/10.1038/s41598-017-02761-6.

38. Steinthorsdottir, V. *et al.* Genetic predisposition to hypertension is associated with preeclampsia in European and Central Asian women. *Nature Communications* **11** (1), 5976 (2020). URL https://doi.org/10.1038/s41467-020-19733-6. https://doi.org/10.1038/s41467-020-19733-6.

39. Kaartokallio, T. *et al.* Gene expression profiling of pre-eclamptic placentae by RNA sequencing. *Scientific Reports* **5** (1), 14107 (2015). URL https://doi.org/10.1038/srep14107. https://doi.org/10.1038/srep14107.

40. Ariff, A., Melton, P. E., Brennecke, S. P. & Moses, E. K. Analysis of the Epigenome in Multiplex Pre-eclampsia Families Identifies SORD, DGKI, and ICA1 as Novel Candidate Risk Genes. *Frontiers in Genetics* **10**, 227 (2019). URL https://www.frontiersin.org/article/10.3389/fgene.2019.00227. https://doi.org/10.3389/fgene.2019.00227.

41. Ma, X., Li, J., Brost, B., Cheng, W. & Jiang, S.-W. Decreased expression and DNA methylation levels of GATAD1 in preeclamptic placentas. *Cellular Signalling* **26** (5), 959–967 (2014). URL http://www.sciencedirect.com/science/article/pii/S0898656814000321. https://doi.org/10.1016/j.cellsig.2014.01.013.

42. Espinosa, C. *et al.* Data-Driven Modeling of Pregnancy-Related Complications. *Trends in Molecular Medicine* (2021). https://doi.org/10.1016/j.molmed.2021.01.007, publisher: Elsevier .

43. Fyhrquist, N. *et al.* Microbe-host interplay in atopic dermatitis and psoriasis. *Nature Communications* **10** (1), 4703 (2019). https://doi.org/10.1038/s41467-019-12253-y .

44. Guenin-Macé, L. *et al.* Dysregulation of tryptophan catabolism at the host-skin microbiota interface in hidradenitis suppurativa. *JCI Insight* **5** (20) (2020). https://doi.org/10.1172/jci.insight.140598, publisher: The American Society for Clinical Investigation .

45. Agus, A., Planchais, J. & Sokol, H. Gut Microbiota Regulation of Tryptophan Metabolism in Health and Disease. *Cell Host & Microbe* **23** (6), 716–724 (2018). https://doi.org/10.1016/j.chom.2018.05.003 .

46. Cuny, H. *et al.* NAD deficiency due to environmental factors or gene–environment interactions causes congenital malformations and miscarriage in mice. *Proceedings of the National Academy of Sciences* **117** (7), 3738 (2020). https://doi.org/10.1073/pnas.1916588117 .

47. Huang, S. *et al.* The putative tumor suppressor Zc3h12d modulates toll-like receptor signaling in macrophages. *Cellular Signalling* **24** (2), 569–576 (2012). https://doi.org/10.1016/j.cellsig.2011.10.011 .

48. Emming, S. *et al.* A molecular network regulating the proinflammatory phenotype of human memory T lymphocytes. *Nature Immunology* **21** (4), 388–399 (2020). https://doi.org/10.1038/s41590-020-0622-8 .

49. Kazmi Nabila *et al.* Hypertensive Disorders of Pregnancy and DNA Methylation in Newborns. *Hypertension* **74** (2), 375–383 (2019). URL https://doi.org/10.1161/HYPERTENSIONAHA.119.12634. https://doi.org/10.1161/HYPERTENSIONAHA.119.12634, publisher: American Heart Association .

50. Fernando, F. *et al.* The idiopathic preterm delivery methylation profile in umbilical cord blood DNA. *BMC Genomics* **16** (1), 736 (2015). URL https://doi.org/10.1186/s12864-015-1915-4. https://doi.org/10.1186/s12864-015-1915-4 .

51. Kaye David M. *et al.* Deficiency of Prebiotic Fiber and Insufficient Signaling Through Gut Metabolite-Sensing Receptors Leads to Cardiovascular Disease. *Circulation* **141** (17), 1393–1403 (2020). URL https://doi.org/10.1161/CIRCULATIONAHA.119.043081. https://doi.org/10.1161/CIRCULATIONAHA.119.043081, publisher: American Heart Association .

52. Sun, S.-C. Non-canonical NF-B signaling pathway. *Cell Research* **21** (1), 71–85 (2011). URL https://doi.org/10.1038/cr.2010.177. https://doi.org/10.1038/cr.2010.177 .

53. Di Stefano, V., Wang, B., Parobchak, N., Roche, N. & Rosen, T. RelB/p52-mediated NF-B signaling alters histone acetylation to increase the abundance of corticotropin-releasing hormone in human placenta. *Science Signaling* **8** (391), ra85 (2015). URL http://stke.sciencemag.org/content/8/391/ra85.abstract. https://doi.org/10.1126/scisignal.aaa9806 .

54. Jie, Z. *et al.* NIK signaling axis regulates dendritic cell function in intestinal immunity and homeostasis. *Nature Immunology* **19** (11), 1224–1235 (2018). URL https:

//doi.org/10.1038/s41590-018-0206-z. https://doi.org/10.1038/s41590-018-0206-z.

55. Richards, A. L. *et al.* Genetic and Transcriptional Analysis of Human Host Response to Healthy Gut Microbiota. *mSystems* **1** (4), e00067–16 (2016). https://doi.org/10.1128/mSystems.00067-16.

56. Kayama, H. *et al.* BATF2 prevents T-cell-mediated intestinal inflammation through regulation of the IL-23/IL-17 pathway. *International Immunology* **31** (6), 371–383 (2019). URL https://doi.org/10.1093/intimm/dxz014. https://doi.org/10.1093/intimm/dxz014.

57. Chehoud, C. *et al.* Complement modulates the cutaneous microbiome and inflammatory milieu. *Proceedings of the National Academy of Sciences* **110** (37), 15061 (2013). URL http://www.pnas.org/content/110/37/15061.abstract. https://doi.org/10.1073/pnas.1307855110.

58. Meisel, J. S. *et al.* Commensal microbiota modulate gene expression in the skin. *Microbiome* **6** (1), 20 (2018). URL https://doi.org/10.1186/s40168-018-0404-9. https://doi.org/10.1186/s40168-018-0404-9.

59. Earley, A. M., Graves, C. L. & Shiau, C. E. Critical Role for a Subset of Intestinal Macrophages in Shaping Gut Microbiota in Adult Zebrafish. *Cell Reports* **25** (2), 424–436 (2018). URL https://www.sciencedirect.com/science/article/pii/S2211124718314554. https://doi.org/10.1016/j.celrep.2018.09.025.

60. Li, Y. *et al.* Tim-3 signaling in peripheral NK cells promotes maternal-fetal immune tolerance and alleviates pregnancy loss. *Science Signaling* **10** (498), eaah4323

(2017). URL http://stke.sciencemag.org/content/10/498/eaah4323.abstract. https://doi.org/10.1126/scisignal.aah4323 .

61. Le Gars, M. *et al.* Pregnancy-Induced Alterations in NK Cell Phenotype and Function. *Frontiers in Immunology* **10**, 2469 (2019). URL https://www.frontiersin.org/article/10.3389/fimmu.2019.02469. https://doi.org/10.3389/fimmu.2019.02469 .

62. Abu-Raya, B., Michalski, C., Sadarangani, M. & Lavoie, P. M. Maternal Immunological Adaptation During Normal Pregnancy. *Frontiers in Immunology* **11**, 2627 (2020). URL https://www.frontiersin.org/article/10.3389/fimmu.2020.575197. https://doi.org/10.3389/fimmu.2020.575197 .

63. Pflitsch, C. *et al.* In-depth characterization of monocyte subsets during the course of healthy pregnancy. *Journal of Reproductive Immunology* **141**, 103151 (2020). URL https://www.sciencedirect.com/science/article/pii/S0165037820300723. https://doi.org/10.1016/j.jri.2020.103151 .

64. Klein, S. L. & Flanagan, K. L. Sex differences in immune responses. *Nature Reviews Immunology* **16** (10), 626–638 (2016). URL https://doi.org/10.1038/nri.2016.90. https://doi.org/10.1038/nri.2016.90 .

65. Kadel, S. & Kovats, S. Sex Hormones Regulate Innate Immune Cells and Promote Sex Differences in Respiratory Virus Infection. *Frontiers in Immunology* **9**, 1653 (2018). URL https://www.frontiersin.org/article/10.3389/fimmu.2018.01653. https://doi.org/10.3389/fimmu.2018.01653 .

66. Guzman-Genuino, R. M., Hayball, J. D. & Diener, K. R. Regulatory B Cells: Dark Horse in Pregnancy Immunotherapy? *Journal of Molecular Biology* **433** (1), 166596

(2021). URL https://www.sciencedirect.com/science/article/pii/S0022283620304551. https://doi.org/10.1016/j.jmb.2020.07.008 .

67. Busse, M. *et al.* Regulatory B Cells Are Decreased and Impaired in Their Function in Peripheral Maternal Blood in Pre-term Birth. *Frontiers in Immunology* **11**, 386 (2020). URL https://www.frontiersin.org/article/10.3389/fimmu.2020.00386. https://doi.org/10.3389/fimmu.2020.00386 .

68. Lima, J. *et al.* Serum markers of B-cell activation in pregnancy during late gestation, delivery, and the postpartum period. *American Journal of Reproductive Immunology* **81** (3), e13090 (2019). URL https://doi.org/10.1111/aji.13090. https://doi.org/10.1111/aji.13090, publisher: John Wiley & Sons, Ltd .

69. Pearson, K. & Henrici, O. M. F. E. Vii. mathematical contributions to the theory of evolution.& iii. regression, heredity, and panmixia. *Philosophical Transactions of the Royal Society of London. Series A, Containing Papers of a Mathematical or Physical Character* **187**, 253–318 (1896). https://doi.org/10.1098/rsta.1896.0007 .

70. Stanton, J. M. Galton, pearson, and the peas: A brief history of linear regression for statistics instructors. *Journal of Statistics Education* **9** (3) (2001). https://doi.org/10.1080/10691898.2001.11910537 .

71. Rodgers, J. L. & Nicewander, W. A. Thirteen ways to look at the correlation coefficient. *The American Statistician* **42** (1), 59–66 (1988). URL http://www.jstor.org/stable/2685263 .

72. Galton, F. I. co-relations and their measurement, chiefly from anthropometric data. *Proceedings of the Royal Society of London* **45** (273-279), 135–145 (1889).

URL https://royalsocietypublishing.org/doi/abs/10.1098/rspl.1888.0082. https://doi.org/10.1098/rspl.1888.0082 .

73. Darwin, C. & Gray, A. *The variation of animals and plants under domestication* Vol. v.2 (1868) (1868). URL https://www.biodiversitylibrary.org/item/84247.
